# The bile acid-gut microbiota-vitamin D axis: new insights into biliary atresia

**DOI:** 10.3389/fcimb.2026.1847110

**Published:** 2026-06-23

**Authors:** Chu Huang, Zongxian Li, Wei Shen, Meilin Luo, Faquan Lin

**Affiliations:** Department of Clinical Laboratory, the First Affiliated Hospital of Guangxi Medical University. Key Laboratory of Clinical Laboratory Medicine of Guangxi Medical University, Education Department of Guangxi Zhuang Autonomous Region, Nanning, Guangxi, China

**Keywords:** biliary atresia, bile acid, gut microbiota, vitamin D, positive feedback loop

## Abstract

Biliary atresia (BA) is a progressive fibroinflammatory disease affecting both intrahepatic and extrahepatic bile ducts. It leads to cholestasis, hepatic fibrosis, and eventually biliary cirrhosis. Although BA is the most common cause of neonatal cholestasis, its exact pathogenesis remains incompletely understood. Emerging evidence suggests that disturbances in bile acid metabolism, gut microbiota dysbiosis, and vitamin D deficiency may play critical roles in the initiation and progression of BA. This review systematically summarizes the dynamic evolution characteristics of bile acid profiles, gut microbiota composition, and vitamin D levels in children with BA across different disease stages. It further explores the potential positive feedback loop effects of the bile acid-gut microbiota-vitamin D axis in the pathogenesis and progression of the disease. Additionally, this review discusses potential diagnostic value and therapeutic strategies based on this axis. Interventions targeting bile acid metabolism, modulating the gut microbiota, and supplementing vitamin D have shown promise in altering the disease course. Despite these advances, further research is needed to elucidate the precise molecular mechanisms underlying this axis in BA. Such knowledge will pave the way for more effective preventive and therapeutic strategies for this complex disease.

## 1 Introduction

Biliary atresia (BA) is a destructive inflammatory and fibroobliterative cholangiopathy of unknown etiology that primarily affects neonates and infants (van Wessel et al., 2021; Hai-Bing et al., 2024; Tam et al., 2024). It is the most common cause of neonatal cholestasis and a leading indication for pediatric liver transplantation (LT) (Feldman and Sokol, 2019). Without timely intervention, affected infants rapidly progress to hepatic fibrosis and end-stage liver disease, with significant mortality (Gupta et al., 2024). Kasai hepatoportoenterostomy (KPE) is the current first-line treatment for BA. However, even with successful surgery, patients remain at high risk for postoperative cholangitis and progressive liver fibrosis. A substantial proportion of these children ultimately develop hepatic failure and require LT (Lynch et al., 2023). Furthermore, intraoperative cholangiography remains the gold standard for diagnosing BA, and reliable non-invasive biomarkers for definitive diagnosis are still lacking (Song et al., 2025). Therefore, there is an urgent clinical need to gain a deeper understanding of the pathogenesis of BA and to explore potential diagnostic and therapeutic strategies.

In recent years, the study of the gut microbiota in neonates with BA has become a focus of intense research. Multiple studies have demonstrated the presence of gut microbiota dysbiosis and disrupted bile acid metabolism in children with BA. These studies have also provided preliminary insights into the correlation between these two factors. Evidence indicates that the gut microbiota regulates bile acid metabolism while also being subject to feedback regulation by bile acids (Chen et al., 2023). Moreover, bile acids facilitate the absorption of vitamin D (Long et al., 2017). The gut microbiota can also influence the synthesis and metabolism of vitamin D, thereby affecting its serum levels (Huang et al., 2019). Conversely, vitamin D can indirectly impact bile acid homeostasis by modulating the gut microbial ecosystem (Yang X. et al., 2022). Research interest in the role of their interaction in children with BA is growing.

Notably, as early as 1979, Kobayashi A et al. (Kobayashi et al., 1979) first reported the presence of 25(OH)D deficiency in children with BA. However, this finding received little attention and was not extensively investigated for several decades. With recent technological advances and more in-depth research, multiple studies have now begun to focus on vitamin D deficiency in children with BA and to explore its underlying causes. Ng J et al. (Ng et al., 2016) and Zhuang P et al. (Zhuang et al., 2019) have subsequently confirmed vitamin D deficiency in children with BA. Nevertheless, the precise mechanisms underlying this deficiency and its intrinsic relationship with hepatic fibrosis remain to be elucidated.

This evidence suggests a complex regulatory relationship among bile acids, the gut microbiota, and vitamin D. They may form a functional axis that contributes to the pathogenesis and progression of BA in children. However, the systematic and functional connections among these three factors and BA have not been fully explored. Therefore, this review proposes a potential theoretical framework centered on the bile acid-gut microbiota-vitamin D axis. Based on existing literature, it provides a systematic overview of BA and examines the dynamic changes in these three factors across different disease stages. It also explores how their positive feedback loop may contribute to disease progression in children with BA. Based on this axis, the review further discusses potential non-invasive diagnostic biomarkers and therapeutic strategies, with the aim of offering new insights for the clinical management of BA. For this review, we searched PubMed, Web of Science, and CNKI up to March 2026 using keywords related to biliary atresia, bile acids, gut microbiota, and vitamin D. Only original articles and reviews in English or Chinese were included. All authors independently screened titles and abstracts, and disagreements were resolved by consensus. The references cited in this review were finally incorporated.

## 2 Overview of biliary atresia

### 2.1 Epidemiology

BA is not only a rare disease of the liver and bile ducts but also a global condition affecting multiple ethnicities. It occurs in infants and is associated with high mortality (de Jong et al., 2023; Hai-Bing et al., 2024). Its incidence varies considerably across geographic regions and ethnic groups (Song et al., 2021a), with overall estimates ranging from approximately 1:5, 000 to 1:20, 000 live births in 2024 (Hai-Bing et al., 2024). The highest incidence is observed in Asia (Antala and Taylor, 2022; Jiang et al., 2024), particularly in East Asian countries where rates can reach 1:3, 000 live births (Kobayashi and Stringer, 2003; Mack, 2007). Reported incidence rates range from 1:5, 000 to 1:9, 000 live births in Shanghai and Hong Kong (Chung et al., 2020), Taiwan (Hsiao et al., 2008), Japan (Nio et al., 2003), and South Korea (Lee et al., 2017). In contrast, incidence rates in Western countries are lower, typically ranging from 1:14, 000 to 1:22, 000 live births (Fanna et al., 2019; Nomden et al., 2021). Even within the same country, ethnic differences exist. For example, in New Zealand, the incidence among Māori children is 1:5, 000 live births, significantly higher than the 1:16, 000 observed in children of European descent (Evans et al., 2018). Additionally, BA appears to have a slight female predominance. Several studies have reported a higher incidence of BA in females compared to males (Mezina and Karpen, 2015; Hopkins et al., 2017; Le et al., 2022). However, epidemiological data remain scarce or even absent in many Western countries, regions, or developing nations.

### 2.2 Etiology

**Figure 1:**
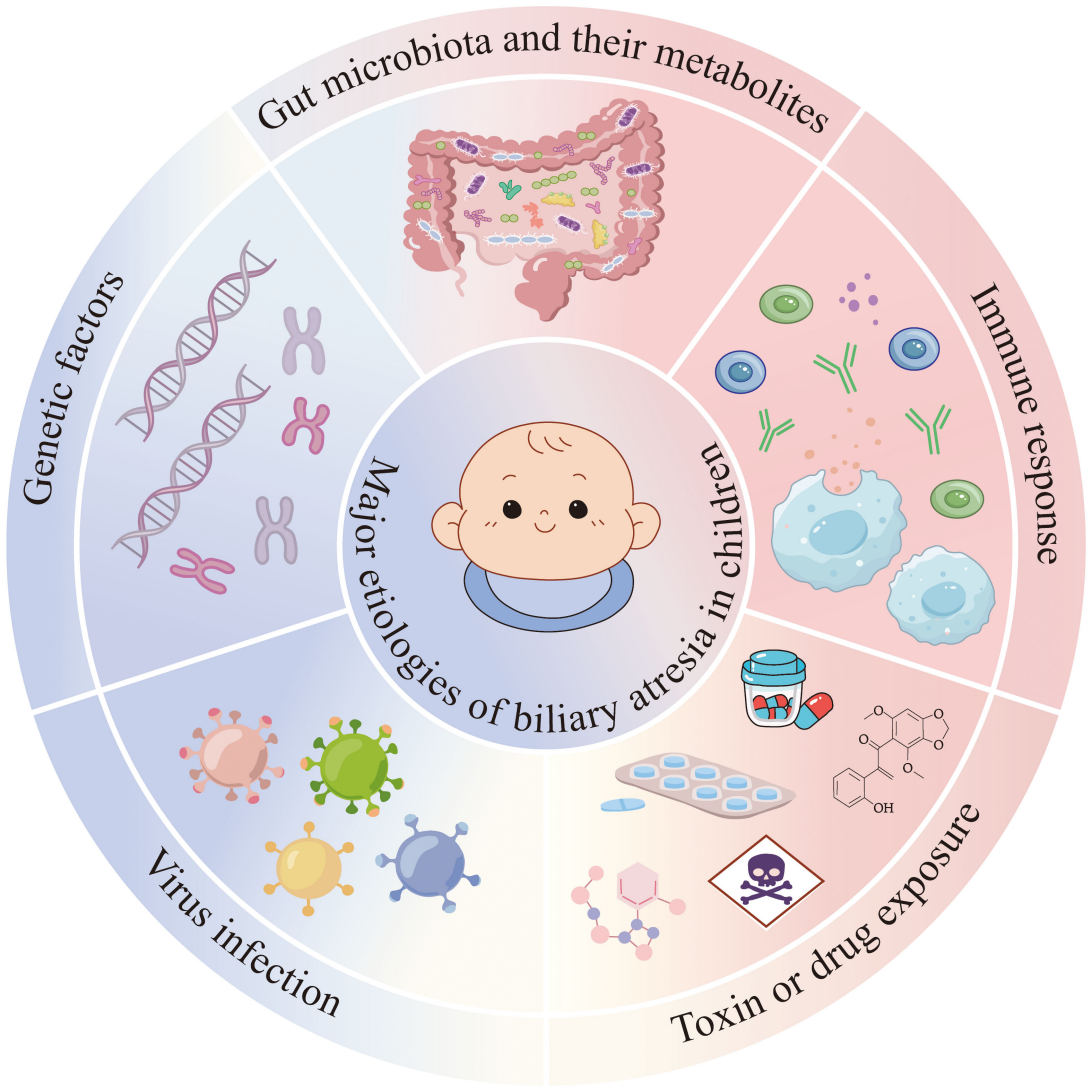
Schematic diagram of the multifactorial etiologies of biliary atresia. Biliary atresia results from a complex interplay of genetic factors, virus infections (e.g., cytomegalovirus, rotavirus), toxins or drug exposure, immune response, gut microbiota and their metabolites. These factors collectively contribute to bile duct injury, inflammation, and fibro-obliteration.

BA is a cholangiopathy characterized by progressive inflammatory and fibroobliterative occlusion of both intrahepatic bile ducts (IHBDs) and extrahepatic bile ducts (EHBDs) (Li TF. et al., 2023). In infants with BA, fibrosis, obstruction, and loss of the EHBD occur within the first few weeks of life (Jansen et al., 2017; Dotan et al., 2022). Without timely diagnosis and treatment, BA rapidly progresses to end-stage liver disease, including cirrhosis and liver failure (Degtyareva et al., 2020). The median survival is 19 months (Lim et al., 2024), and most affected children die within the first two years of life (Hartley et al., 2010). Presently, the pathogenesis of BA remains incompletely understood. Its etiology is considered to be heterogeneous, complex, and multifactorial (Davenport et al., 2021). Currently, the major proposed etiologies of BA include genetic factors, viral infections, toxin or drug exposure, and immune responses (**Figure 1**). Evidence indicates that BA is not caused by a single factor but rather results from the interplay of multiple complex variables and factors (Petersen and Davenport, 2013). These factors may damage the biliary epithelium, leading to bile duct developmental defects, and trigger inflammatory responses and secondary autoimmune reactions in biliary epithelial cells, thereby inducing biliary injury and promoting fibro-obliteration of the bile ducts (Mack et al., 2005; Barnes et al., 2009; Walesky and Goessling, 2016; Averbukh and Wu, 2018). This process ultimately leads to destructive EHBD lesions as a common endpoint (Hartley et al., 2009). Emerging research suggests that an imbalance of the gut microbiota and their metabolites may represent a potential etiological factor in BA (Feng et al., 2024a). Interestingly, studies have found that vitamin D deficiency impacts the composition of major gut bacterial phyla in children, raising the possibility of a link to the risk of developing BA (Singh et al., 2022). However, the precise molecular mechanisms by which these factors interact and drive disease progression remain elusive, and further investigation is needed to establish causal links. Therefore, a thorough understanding of the etiology and pathogenesis of BA is essential for its early diagnosis and prevention, and holds substantial practical significance for developing effective treatment strategies.

## 3 Bile acid profile in biliary atresia

### 3.1 Synthesis and physiological functions of bile acids

Bile acids are the primary metabolic components of bile (Yang M. et al., 2021). In humans, total bile acids (TBA) are categorized by origin into primary and secondary bile acids, and by chemical structure into unconjugated and conjugated forms. Primary bile acids are synthesized from cholesterol in the liver, mainly via the classical or alternative pathway, involving a series of enzymatic oxidation reactions (Yu et al., 2023). The classical pathway is initiated by the rate-limiting enzyme cholesterol 7α-hydroxylase (CYP7A1). Working in concert with sterol 12α-hydroxylase (CYP8B1) and mitochondrial sterol 27-hydroxylase (CYP27A1), it produces cholic acid (CA) and a small quantity of CDCA (Yu et al., 2023). The CYP27A1-initiated alternative pathway generates the majority of CDCA (Jia et al., 2018). Primary bile acids synthesized in the liver are conjugated with glycine or taurine to form conjugated bile acids (Agolino et al., 2024). Once secreted into the small intestine with bile, they undergo enzymatic modification by the gut microbiota, notably 7α-dehydroxylation, which generates secondary bile acids such as lithocholic acid (LCA) and deoxycholic acid (DCA) (Funabashi et al., 2020).

Bile acids not only facilitate lipid emulsification and promote intestinal absorption of lipids and lipophilic vitamins, but also function as potent signaling molecules (Long et al., 2017; Thibaut and Bindels, 2022; Sutton et al., 2024). They help maintain bile acid homeostasis and regulate other metabolic processes by activating host receptors related to bile acids, including the farnesoid X receptor (FXR), the vitamin D receptor (VDR), and the Takeda G protein-coupled receptor 5 (TGR5) expressed on the cell surface (Long et al., 2017; Fiorucci et al., 2024). Among these, the FXR-mediated negative feedback mechanism is essential for bile acid homeostasis (Sayin et al., 2013). Elevated bile acid levels activate both the hepatic FXR-small heterodimer partner (SHP) signaling axis and the ileal FXR-fibroblast growth factor 19 (FGF19)-c-Jun N-terminal kinase (JNK) pathway, thereby suppressing CYP7A1 enzyme activity in the liver to reduce bile acid synthesis (Xiang et al., 2021; Yan et al., 2022; Hong et al., 2023). Current state of the art techniques for studying bile acid modification and physiological functions include ultra performance liquid chromatography tandem mass spectrometry (UPLC-MS/MS) (Feng et al., 2024b; Wang et al., 2024), metagenomic sequencing, reverse metabolomics, culturomics (microbiota co-cultured with BA substrates), source tracking tools (such as MetOrigin and microbeMASST), chemoproteomic profiling of bile acid-metabolizing enzymes, and organoid models (Zhang et al., 2025).

### 3.2 Alterations in bile acid metabolism associated with biliary atresia

Multiple studies have demonstrated that alterations in the bile acid profile are closely associated with the initiation and progression of biliary atresia. Moreover, these changes exhibit dynamic and characteristic evolution throughout the course of the disease in affected children (**Table 1**).

**Table 1:** Bile acid profile alterations at different stages of biliary atresia.

Patient or model	Method	Bile acids	Year	Ref
Biliary atresia without treatment				
7 BDL mice and 7 HC mice	Liquid Chromatography - Tandem Mass Spectrometry Method	12-ketolithocholic acid, 3-dehydrocholic acid, alpha-muricholic acid, beta-muricholic acid, cholic acid, deoxycholic acid, hyocholic acid, lithocholic acid and ursodeoxycholic acid↓↓	2024	Yajun L (Yajun, 2024)
46 BA patients and 38 HC	Targeted metabolomics	Steroid↑↑, primary bile acids↓↓	2022	Yang T et al. (Yang T. et al., 2022)
16 BA (early stage, age < 3 months) patients and 16 HC;16 BA (later stage, age < 3 years) patients and 10 HC	Untargeted metabolomics	Glycoursodeoxycholic aci and taurousodeoxycholic acid↑↑, nor cholic acid, cholic acid, beta-muricholic acid, allocholic acid, taurohyodeoxycholic acid, 3beta-cholic acid, 3-dehydrocholic acid, hyocholic acid, 6, 7-diketolithocholic acid, 3beta-ursodeoxycholic acid, dehydrocholic acid, 7-ketodeoxycholic acid, alpha-muricholic acid, taurocholic acid and taurolithocholate↓↓	2021	Song W et al. (Song et al., 2021a)
8 BDL mice, 8 BDL mice with diphtheria toxin, 6 sham surgery mice and 6 sham surgery mice with diphtheria toxin	Ultra-high performance liquid chromatography-triple quadrupole mass spectrometry	Primary bile acids and Total bile acids↑↑	2021	Xinbei T et al. (Xinbei et al., 2021)
27 BA patients, 34 HC and 23 non-BA cholestasis	Ultra-high performance liquid chromatography-tandem mass spectrometry	Glycine cholic acid and glycine cholic acid/chenodeoxycholic acid↑↑, chenodeoxycholic acid↓↓	2020	Mingming L (Mingming, 2020)
Biliary atresia with KPE				
8 BA patients post-KPE and 8 BA patients non-KPE	Ultraperformance liquid chromatography/tandem mass spectrometry	Cholic acid, chenodeoxycholic acid, β-muricholic acid and tauro α-muricholate↑↑	2021	Song W et al. (Song et al., 2021b)
Biliary atresia with LT				
12 BA patients post LT 24+ months and 19 HC	Targeted metabolomic	Secondary bile acid↑↑	2023	Waldner B et al. (Waldner et al., 2023)

‘↑’ indicates increase and ‘↓’ indicates decrease compared with controls. BA, biliary atresia; HC, healthy control; BDL, bile duct ligation; KPE, Kasai portoenterostomy; LT, liver transplantation.

In untreated children with BA, disruption of normal biliary drainage reduces bile flow from the liver to the intestine, leading to impaired bile acid metabolism and an abnormally altered bile acid profile (Lynch et al., 2023). These changes feature markedly elevated levels of total and primary bile acids in both serum and liver, coupled with reduced secondary bile acids and increased sterol levels (Xie et al., 2021; Yang T. et al., 2022). In feces, the abundance of bile acids is also significantly decreased (Zhou et al., 2015). Specifically, a study analyzing bile acid profiles in liver and colonic contents from a mouse model of BA similarly reported significantly increased levels of total and primary bile acids in the liver (Xinbei et al., 2021).

As BA progresses to hepatic fibrosis, TBA levels increase (Wang et al., 2024). Bile acid profiling reveals elevated levels of glycochenodeoxycholic acid (GCDCA), taurochenodeoxycholic acid (TCDCA), glycocholic acid (GCA), and taurocholic acid (TCA) compared to healthy controls (Feng et al., 2024b). Moreover, these components may further increase as cirrhosis develops (Han et al., 2022).

A bile acid profiling study in children with BA after KPE showed significantly elevated levels of GCA, whereas CDCA was markedly reduced (Mingming, 2020). Another study reported that following KPE, children with BA continued to exhibit significantly elevated levels of primary bile acids, including CA, CDCA, β-muricholic acid, and tauro-α-muricholate (Song et al., 2021b). Marked increases in certain secondary bile acids were also observed, such as α-hydroxydeoxycholic acid (HDCA) (Song et al., 2021b). These findings suggest that after KPE, partial restoration of bile flow leads to a decline in some primary bile acids and an increase in certain secondary bile acids, which together help restore total bile acid levels toward normal. Nevertheless, TBA levels often do not fully normalize, consistent with the subsequent risk of recurrent cholangitis and progressive hepatic fibrosis frequently seen in these patients.

After LT in children with BA, enterohepatic circulation is re-established, followed by a marked increase in secondary bile acid concentrations within two years (Waldner et al., 2023).

## 4 Gut microbiota in biliary atresia

### 4.1 The gut microbiota in children

The human gut microbiota is regarded as another human organ and the second human genome (Ogbu et al., 2020; Song et al., 2021b). The human gut microbiota consists of approximately 10^13^ to 10^14^ bacteria (Nicholson et al., 2012). The four dominant phyla are *Bacteroidetes*, *Firmicutes*, *Proteobacteria*, and *Actinobacteria*, with over 90% of the gut microbiota belonging to the phyla *Bacteroidetes* and *Firmicutes* (Sun et al., 2022; Wang et al., 2024).

The diversity, composition, and metabolic functions of the infant gut microbiota undergo dynamic physiological succession shaped by age and environmental factors (Hollister et al., 2015). Gut microbiota colonization in infants follows a progressive temporal pattern (Rager and Zeng, 2023). This process begins during gestation, with bacterial colonization occurring immediately after birth (Nicholson et al., 2012). Early colonizers include aerotolerant *Bifidobacterium*, microaerophilic *Lactobacillus*, and facultative Enterobacteriaceae species (Rager and Zeng, 2023). By the age of 4 months, the infant gut microbiota is typically dominated by *Bifidobacterium*, *Lactobacillus*, and *Veillonella* (Backhed et al., 2015). At around 6 months of age, the introduction of fiber-containing foods promotes the expansion of fiber-fermenting genera such as *Clostridium*, *Akkermansia*, *Bacteroides*, and *Ruminococcus*, while the levels of *Escherichia* and *Staphylococcus* decline (Bergström et al., 2014; Davis et al., 2017; Laursen et al., 2017). Overall, during full-term development, neonates are typically colonized by beneficial bacteria, including *Bifidobacterium* and *Bacteroides* (Saturio et al., 2021). The microbiota continues to evolve with age and reaches an adult-like configuration within the first 1 to 3 years of life (Koenig et al., 2011; Bergström et al., 2014). In addition, the composition of the infant gut microbiota is shaped by multiple factors, including whether delivery is vaginal or cesarean, whether diet is breast milk or formula, as well as bile secretion, viral infection, and toxin exposure (Hasan and Yang, 2019; Mitchell et al., 2020; Wang et al., 2023).

The gut microbiota plays an essential role in maintaining human health, with functions encompassing the facilitation of digestion, nutrient absorption, vitamin synthesis, and immune modulation (Goulet, 2015; Waldner et al., 2023). Additionally, it contributes to the maintenance of intestinal barrier integrity, protection against pathogenic microorganisms, and involvement in bile acid synthesis and metabolism (Garzarella et al., 2022; Aggeletopoulou et al., 2023). Recent advances in omics technologies have enabled the development of novel tools for profiling gut microbial composition and function. These include 16S rRNA gene sequencing (Yang T. et al., 2022; Liu Y. et al., 2024), metagenomic sequencing (Song et al., 2021b), and fecal culture-based identification using an albumin-based semi-automated microbial identification system (Cejun et al., 2022).

### 4.2 Alterations in the gut microbiota associated with biliary atresia

In recent years, a growing body of evidence has highlighted that alterations in the composition and function of the gut microbiota are closely associated with the development and progression of biliary atresia. The diversity, composition, and function of the gut microbiota vary considerably across different stages of the disease in children with BA. Delineating these specific alterations provides valuable insights into the disease course and informs the development of potential therapeutic strategies.

#### 4.2.1 Gut microbiota alterations in BA without treatment

Abnormal changes in the diversity, composition, and structure of the gut microbiota are collectively referred to as gut microbiota dysbiosis (Tangestani et al., 2020). Studies investigating the gut microbiota in infants with BA have consistently shown that untreated children with BA exhibit marked dysbiosis, with significant differences compared to healthy infants or those with non-BA cholestasis (**Table 2**). This dysbiosis is characterized by reduced microbial diversity, an increased abundance of pathogenic bacteria, a decreased abundance of beneficial bacteria, along with pronounced alterations in microbial function. Multiple studies have consistently reported that the gut microbiota composition in BA is markedly disturbed compared to control groups (Wang et al., 2020; Song et al., 2021a; van Wessel et al., 2021; Yang T. et al., 2022; Liu F. et al., 2024). At the phylum level, *Proteobacteria* are enriched, while *Bacteroidetes* and *Actinobacteria* are decreased. At the genus level, opportunistic or potentially pathogenic bacteria are commonly enriched, including *Klebsiella*, *Streptococcus*, *Veillonella*, and *Enterococcus*. In contrast, butyrate-producing beneficial genera are significantly reduced, such as *Bifidobacterium*, *Faecalibacterium*, and *Blautia*. Notably, *Klebsiella*, *Streptococcus*, *Veillonella*, and *Enterococcus* act as key pathogens driving disease progression in both early and later stages of BA. Besides, these pathogenic genera become even more enriched as the disease progresses to later stages (Song et al., 2021a). Furthermore, the abundance of *Veillonella atypica* differs significantly between early and late untreated BA, with markedly higher levels observed in the late stage (Song et al., 2021a). This increase may be linked to the reduced intestinal bile acid levels resulting from more severe biliary obstruction in late BA (Wang et al., 2020). Moreover, the elevated abundance of *V. atypica* may in turn contribute to liver injury (Song et al., 2021b). In addition to compositional disturbances, functional alterations of the gut microbiota have also been observed in children with BA, including enhanced pathogenic functions such as tryptophan metabolism, polysaccharide biosynthesis, and bacterial secretion systems (Sun et al., 2022). While the above alterations represent common features of gut microbiota dysbiosis in BA, some differences exist across different studies. For instance, some studies have reported enrichment of *Lactobacillus* or *Fusobacterium* (Xu et al., 2023; Jain et al., 2024). This may be associated with study populations, disease stages, or analytical methods, suggesting that the specific manifestations of dysbiosis in BA may be heterogeneous.

**Table 2:** Gut microbiota alterations in biliary atresia without treatment.

Patient or model	Method	Diversity	Dysbiosis of the microbes	Functional profiles	Year	Ref
12 BA patients, 8 HC and 8 non-BA cholestasis	Fecal 16S rRNA gene sequencing	No change	Proteobacteria, unclassified Enterobacteriaceae*, Bacteroides* and *Dialister*↑↑, Firmicutes↓↓	NA	2025	Azizah N et al. (Azizah et al., 2025)
26 BA patients, 50 HC and 37 non-BA cholestasis	Fecal 16S rDNA gene sequencing	Reduced	*Bifidobacterium, Enterococcus, Limosilactobacillus reuteri, Blautia, Agathobacter rectalis, Veillonella, Streptococcus, Phocaeicola plebeius, Rothia mucilaginosa* and *Bacteroides*↓↓	NA	2024	Liu Y et al. (Liu Y. et al., 2024)
33 BA patients, 17 HC and 19 non-BA cholestasis	Fecal 16S rRNA gene sequencing	No change	*Enterococcus*, *Clostridium*, *Fusobacterium*, and *Pseudomonas*↑↑, *Bifidobacterium*, *Haemophilus*↓↓	NA	2024	Jain V et al. (Jain et al., 2024)
31 BA patients and 20 HC	Fecal 16S rRNA gene sequencing	Reduced	Firmicutes, Fusobacteria*, Streptococcus, Klebsiella and Veillonella*↑↑, Actinobacteria*, Bifidobacterium, Ruminococcus and Rothia*↓↓	NA	2024	Liu F et al. (Liu F. et al., 2024)
26 BA patients, 50 HC and 37 non-BA cholestasis	Fecal 16S rDNA gene sequencing	Reduced	*Bifidobacterium longum*↑↑*, Bifidobacterium, Enterococcus, Collinsella and Staphylococcus*↓↓	NA	2024	Yajun L (Yajun, 2024)
7 BDL mice and 7 HC mice	Fecal 16S rDNA gene sequencing	No change	Bacteroides, unidentified_Ruminococcaceae, [Eubacterium]_siraeum_group, UBA1819, Family_XIII_UCG-001, *Paludicola*↑↑, Patescibacteria, Desulfobacterota, Actinomycetota, *Ligilactobacillus*, Candidatus_Saccharimonas, *Desulfovibrio, Enterobacter, Parvibacter, Bilophila*, Candidatus and Stoquefichus↓↓	NA	2024	Yajun L (Yajun, 2024)
11 BA patients, 6 HC and 6 non-BA cholestasis	Fecal 16S rRNA gene sequencing	Reduced	*Proteobacteria, Streptococcus, Lactobacillus* and *Klebsiella*↑↑, Bacteroidetes phylum, *Escherichia*, *Enterococcus*, *Eubacterium*, *Bacteroides* and *Eggerthella*↓↓	NA	2023	Xu X et al. (Xu et al., 2023)
46 BA patients and 38 HC	Fecal 16S rRNA gene sequencing	Reduced	*Escherichia-Shigella*, *Streptococccus* and *Veillonella*↑↑, *Actinomyces*, *Faecalibacterium*, *Agathobacter*, *Blautia*, and *Eggerthella* ↓↓	NA	2022	Yang T et al. (Yang T. et al., 2022)
17 BA patients and 8 non-BA cholestasis	Fecal 16S rDNA gene sequencing	No change	*Enterococcus*, *Ralstonia* and Nitriliruptoraceae↑↑, Proteobacteria, Bacteroidetes and Firmicutes↓↓	NA	2022	Sun X et al. (Sun et al., 2022)
BDL rat model (n=6-7), BDL rat model with obeticholic acid (n=6-7) and HC rat model (n=6-7)	Fecal 16S rDNA MiSeq sequencing	Reduced	Bacteroidetes, Lachnospiraceae NK4A136 group, *Alistipes*, Eubacterium_ruminantium_group, *Intestinimonas*, *Ruminococcus_1* and Ruminococcaceae_NK4A214_group↑↑, Phylum Firmicutes and *Lactobacillus*↓↓	NA	2022	Yan M et al. (Yan et al., 2022)
8 BDL mice, 8 BDL mice with diphtheria toxin, 6 sham surgery mice and 6 sham surgery mice with diphtheria toxin	Fecal 16S rRNA gene sequencing	NA	*Blautia, Streptococcus, Christensenella* and *Aggregatibacter*↑↑, *Prevotella spp*↓↓	NA	2021	Xinbei T et al. (Xinbei et al., 2021)
16 BA (early stage, age < 3 months) patients and 16 HC	Metagenomic sequencing	Reduced	Proteobacteria, *Klebsiella*, *Streptococcus*, *Veillonella* and *Enterococcus*↑↑, Actinobacteria, Verrucomicrobia, *Bifidobacterium* and *Blautia*↓↓	Capsular polysaccharide transport system, LPS biosynthesis, adhesins, adhesion protein transport system, bacterial secretion systems, ketone body biosynthesis, polyamine biosynthesis, GABA biosynthesis, aromatic amino acid metabolism, and branched-chain amino acid metabolism (isoleucine biosynthesis) ↑↑	2021	Song W et al. (Song et al., 2021a)
16 BA (later stage, age < 3 years) patients and 10 HC		Reduced	Proteobacteria, *Klebsiella*, *Streptococcus*, *Veillonella* and *Enterococcus*↑↑, Bacteroidetes and Verrucomicrobia↓↓			
12 BA patients and 6 HC	Fecal 16S rRNA gene sequencing	No change	*Streptococcus* ↑↑, Bifidobacteriaceae and Lachnospiraceae↓↓	NA	2021	van Wessel D et al. (van Wessel et al., 2021)
43 BA patients and 22 HC	Fecal 16S rRNA gene sequencing	Reduced	Firmicutes, Proteobacteria*, Streptococcus*, *Klebsiella*, *Haemophilus*↑↑, *Bifidobacterium*, *Bacteroides and Lactobacillus*↓↓	NA	2021	Bolin C (Bolin, 2021)
34 BA patients and 34 HC	Fecal 16S rRNA gene sequencing and metagenomic sequencing	Reduced	Proteobacteria, *Streptococcus*, *Klebsiella* and *Enterococcus*↑↑, Bacteroidetes, *Bifidobacterium*, *Faecalibacterium*, *Faecalibacterium*, Lachnospiraceae, Clostridium XIVa *and Blautia*↓↓	NA	2020	Wang J et al. (Wang et al., 2020)
11 BA patients and 10 HC	Fecal 16S rRNA gene sequencing	Reduced	Firmicutes and *Veillonella*↑↑, Bacteroidetes phylum*, Bacteroides, Clostridium*↓↓	NA	2017	Meilin H (Meilin, 2017)

‘↑’ indicates increase and ‘↓’ indicates decrease compared with controls. ‘NA’ denotes ‘not applicable’, indicating that data for a specific item are missing or irrelevant.BA, biliary atresia; HC, healthy control; BDL, bile duct ligation.

#### 4.2.2 Gut microbiota alterations in biliary atresia after KPE

After KPE, biliary drainage is restored in children with BA, accompanied by resolution of gut microbiota dysbiosis (**Table 3**). This improvement is marked by increased microbial diversity and enrichment of potentially beneficial genera, including *Bifidobacterium*, *Faecalibacterium*, *Actinomyces*, and *Lactobacillus* (Song et al., 2021b). Notably, *V. atypica*, which is the species most strongly associated with liver injury, declines after surgery (Song et al., 2021b). Consistently, Elaine Chen YF et al. (Elaine Chen et al., 2020) observed abundant *Bifidobacterium* in the stool of patients whose liver function and jaundice progressively improved within six weeks after KPE. Furthermore, Cejun F et al. (Cejun et al., 2022) reported that within six months after KPE, intestinal levels of *Bifidobacterium* and *Lactobacillus* were significantly higher than preoperative values, whereas *Faecalibacterium* levels were markedly reduced.

**Table 3:** Gut microbiota alterations in biliary atresia after KPE.

Patient	Method	Diversity	Dysbiosis of the Microbes	Functional profiles	Year	Ref
33 BA patients post-KPE, 17 HC and 19 non-BA cholestasis	Fecal 16S rRNA gene amplicon sequencing	Increased	*Streptococcus* and *Fusobacterium*↑↑, *Bifidobacterium*, *Dorea*, *Blautia* and *Oscillospira*↓↓	NA	2024	Jain V et al. (Jain et al., 2024)
33 BA patients (before and after KPE)	Fecal 16S rRNA gene amplicon sequencing	Increased	*Enterococcus, Pseudomonas, Fusobacterium, Actinomyces*, and *Haemophilus*↑↑, *Streptococcus*↓↓	NA	2024	Jain V et al. (Jain et al., 2024)
55 BA patients, 19 HC and 21 non-BA cholestasis	Fecal 16S rRNA gene sequencing	Increased	*Enterococcus↑↑, Bifidobacterium↓↓*	NA	2023	Jain V et al. (Jain et al., 2023)
40 BA patients (before and after KPE)	Fecal culture	NA	*Bifidobacterium* and *Lactobacillus*↑↑, *Faecalibacterium*↓↓	NA	2022	Cejun F et al. (Cejun et al., 2022)
8 BA patients post-KPE and 8 BA patients non-KPE	Fecal 16S rRNA gene sequencing, Metagenomic sequencing	Increased	*Bacteroides* spp.*, Prevotella* spp.*, Barnesiella* spp.*, Parabacteroides* spp.*, Heliobacterium* spp.*, Erysipelatoclostridium* spp. and *Diaporthe spp*↑↑, *Veillonella atypica*↓↓	Pyridoxal biosynthesis and Riboflavin biosynthesis↑↑	2021	Song W et al. (Song et al., 2021b)
30 BA patients and 23 HC	Fecal 16S rRNA gene sequencing	NA	*Enterococcus↑↑*	NA	2021	Orłowska E et al. (Orłowska et al., 2021)

‘↑’ indicates increase and ‘↓’ indicates decrease compared with controls. ‘NA’ denotes ‘not applicable’, indicating that data for a specific item are missing or irrelevant.BA, biliary atresia; HC, healthy control; KPE, Kasai portoenterostomy.

However, the improvement in gut microbiota dysbiosis following KPE in children with biliary atresia may be transient and insufficient to fully restore microbial balance (Waldner et al., 2023). Studies have shown that the gut microbiota remains disrupted in children with BA even after KPE (Wang et al., 2020). This may be related to the rate of jaundice resolution following KPE and the depletion of beneficial bacteria caused by postoperative antibiotic therapy (van Wessel et al., 2021). Another possible explanation is that the KPE procedure itself temporarily disrupts the intestinal barrier, leading to increased intestinal permeability that allows pathogenic bacteria to enter the portal vein (Waldner et al., 2023). Specifically, opportunistic pathogens remain persistently enriched, such as *Enterococcus*, *Streptococcus*, *Pseudomonas*, and *Haemophilus*, while potentially beneficial genera become further depleted including *Bifidobacterium*, *Dorea*, *Blautia*, and *Oscillospira*. These alterations are associated with poor resolution of jaundice, the development of cholangitis and an increased need for LT (Jain et al., 2024). Jain V et al. (Jain et al., 2024) found that *Blautia* abundance correlated inversely with liver disease severity, whereas *Bifidobacterium* abundance correlated inversely with fibrotic biomarkers. These observations suggest that sustained depletion of *Blautia* and *Bifidobacterium* is closely associated with the progression of BA to serious liver disease and liver fibrosis.

Following KPE, the functional capacity of the gut microbiota in children with BA also undergoes marked alterations, exemplified by significant enrichment of pyridoxal biosynthesis and riboflavin biosynthesis (M00124 and M00125) (Song et al., 2021b).

#### 4.2.3 Gut microbiota alterations in biliary atresia after LT

LT represents the definitive treatment for biliary atresia, as it restores biliary flow and re-establishes normal digestive physiology. To a certain extent, this intervention markedly improves the composition and function of the gut microbiota in children with BA, although the microbiota does not fully return to the levels observed in healthy children (**Table 4**) (Wei et al., 2021). In the early period after LT, children with BA face an elevated risk of infection (Song et al., 2021c). The antibiotics administered during this phase deplete potentially beneficial gut bacteria, resulting in microbiota dysbiosis (Song et al., 2021c). Accordingly, alpha diversity of the gut microbiota is significantly reduced at three months post-transplantation (Waldner et al., 2023). What’s more, studies by Song W et al. (Song et al., 2021c) and Waldner B et al. (Waldner et al., 2023) have characterized the gut microbiota in children with BA after LT. Their findings show that by two years post-transplantation, alpha diversity approaches that of healthy controls, and the overall microbial composition becomes comparable between the two groups, indicating a shift in the gut microbiota of LT recipients toward that of healthy individuals.

**Table 4:** Gut microbiota alterations in biliary atresia after LT.

Patient	Method	Diversity	Dysbiosis of the microbes	Functional profiles	Year	Ref
12 BA patients post LT 24+ months and 19 HC	Fecal 16S-rRNA-sequencing	No change	*Klebsiella and Enterobacteroidales*↑↑, *Clostridiales* and *Bifidobacteriales*↓↓	Bile acid hydrolase↑↑	2023	Waldner B et al. (Waldner et al., 2023)
16 BA pre-LT, 14 BA post-LT (6 non-KPE, 8 with KPE) and 10 HC	Metagenomic sequencing	No change	*Roseburia, Blautia, Clostridium, Akkermansia*, and *Ruminococcus*↑↑, *Serratia, Enterobacter, Morganella, Skunalikevirus*, and *Phifllikevirus*↓↓	Lipopolysaccharide metabolism, Multidrug resistance, Polyamine biosynthesis, GABA biosynthesis, and EHEC/EPEC pathogenicity signature↑↑	2021	Song W et al. (Song et al., 2021c)
16 BA patients (before and after LT) and 10 HC	Metagenomic sequencing	No change	*Roseburia, Eubacterium, Blautia, Clostridium* and *Akkermansia*↑↑	Amino acid metabolism, Lipid metabolism, Energy metabolism, Carbohydrate metabolism, Metabolism of cofactors and vitamins↑↑, Membrane transport, Infectious diseases: bacterial, Drug resistance, Glycan biosynthesis and metabolism, Immune diseases↓↓	2021	Wei S et al. (Wei et al., 2021)
12 BA pre-LT, 12 BA post-LT and 22 HC	Fecal 16S rRNA gene sequencing	Increased	Firmicutes↑↑, Actinobacteria and Bacteroidetes↓↓	NA	2021	Bolin C (Bolin, 2021)

‘↑’ indicates increase and ‘↓’ indicates decrease compared with controls. ‘NA’ denotes ‘not applicable’, indicating that data for a specific item are missing or irrelevant. BA, biliary atresia; HC, healthy control; LT, liver transplantation.

Meanwhile, Wei S et al. (Wei et al., 2021) reported that the gut microbiota of children with BA before LT was dominated by opportunistic pathogens. Six months after LT, the abundance of these pathogens had significantly declined, whereas that of short-chain fatty acid (SCFA)-producing genera had markedly increased, including *Roseburia*, *Eubacterium*, *Blautia*, *Clostridium*, and *Akkermansia* (Wei et al., 2021). This shift reflects an overall trend toward restoration and stabilization of the intestinal microecology in these children. However, differences between the post-LT group and healthy controls persisted, with significantly higher abundances of *Klebsiella* and *Enterobacterales*, as well as notably lower levels of *Clostridiales* and *Bifidobacteriales* in children after LT (Wei et al., 2021; Waldner et al., 2023). These findings suggest that the composition of the gut microbiota in children with BA after LT has not yet reached the level of that in healthy controls.

Functionally, the core metabolic capacity of the gut microbiota is substantially enhanced in children with BA after LT, with upregulation of pathways involved in amino acid metabolism, lipid metabolism, energy metabolism, carbohydrate metabolism, as well as cofactors metabolism and vitamin metabolism (Wei et al., 2021). Conversely, pathways linked to bacterial infection, immune system disorders, and drug resistance are downregulated (Wei et al., 2021). It is notable that the activity of bile salt hydrolase (BSH), a key enzyme in bile acid metabolism, increases after LT, helping to restore the normal enterohepatic circulation of bile acids in these children (Waldner et al., 2023). However, certain potentially harmful functions persist. Pathways related to lipopolysaccharide metabolism, multidrug resistance, polyamine biosynthesis, GABA biosynthesis, and EHEC/EPEC pathogenicity remain enriched in a subset of children with BA after LT (Song et al., 2021c). These observations suggest that the risk of inflammation or drug resistance may persist in these patients.

## 5 Vitamin D and biliary atresia

### 5.1 Vitamin D metabolism and physiological functions in humans

Vitamin D is a fat-soluble vitamin that is synthesized upon skin exposure to ultraviolet light and obtained through dietary intake (Huang et al., 2019). Diet serves as the primary source of vitamin D. Once absorbed, vitamin D is first hydroxylated in the liver by CYP2R1 and CYP27A1 to form 25-hydroxyvitamin D (25(OH)D, also referred to as 25(OH)D_3_) (Song et al., 2025). This inactive metabolite circulates throughout the body and is stored mainly in the liver and adipocytes (Shirwaikar Thomas et al., 2021). 25(OH)D itself cannot act directly on target tissues. It must be transported via the bloodstream to the kidneys, where it undergoes a second hydroxylation by the enzyme 25-hydroxyvitamin D-1α-hydroxylase (CYP27B1) (Sun et al., 2021). This step converts it into 1, 25-dihydroxyvitamin D (1, 25(OH)_2_D, also known as 1, 25(OH)_2_D_3_), which is the final biologically active form of vitamin D (Sun et al., 2021). Vitamin D metabolites, including 25(OH)D and 1, 25(OH)_2_D, enter the intestine together with bile acids and are reabsorbed in the terminal ileum (Tokita et al., 1991). Moreover, the gut microbiota also shapes 1, 25(OH)_2_D metabolism through the production of specific enzymes and metabolites, as well as by modulating microbiota-associated vitamin metabolic pathways. Certain intestinal bacteria possess the capacity to degrade or transform steroids and may contribute to the hydroxylation of 1, 25(OH)_2_D, thereby affecting its conversion to the active form and its bioavailability (Szaleniec et al., 2018). Beneficial bacteria capable of producing BSH, such as *Lactobacillus* and *Bifidobacterium*, may facilitate the conversion of vitamin D to its active form or enhance its bioavailability (Hou et al., 2025).

Serum 25(OH)D reflects the body’s vitamin D stores, whereas 1, 25(OH)_2_D is the active form that mediates the biological actions of vitamin D (Wang et al., 2025). The physiological functions of vitamin D are mediated through the binding of 1, 25(OH)_2_D to the ubiquitously expressed vitamin D receptor (VDR). VDR belongs to the nuclear hormone receptor superfamily and is expressed in approximately 30 different tissues, including the intestine and fetal tissues (Zhang et al., 2013). 1, 25(OH)_2_D directly activates VDR, subsequently forms a 1, 25(OH)_2_D-RXR-VDR complex by binding to VDR in the cytoplasm and heterodimerising with the retinoic acid X receptor (RXR) in the nucleus (Adams and Hewison, 2010). This complex further binds to vitamin D response elements (VDREs) within the regulatory regions of target genes, thereby activating the expression of multiple genes, including apoptotic and inflammatory-related genes such as Bcl-2 and NF-κB, along with calcium channel genes such as TRPV6 and fibroblast growth factor 23 (FGF23) (Haussler et al., 2011; Zhang et al., 2012; Zeitelhofer et al., 2017). This subsequently contributes to physiological activities such as alleviating oxidative stress, regulating immune and inflammatory responses, promoting apoptosis, regulating the metabolism and absorption of calcium and phosphorus in the gut to maintain bone health, as well as modulating the growth and differentiation of tissue cells (Matta Reddy et al., 2022; Voland et al., 2022). Moreover, vitamin D plays a vital role in preventing liver fibrosis (Tzilas et al., 2019). It can suppress the proliferation of HSCs, as well as reduce the expression of collagen and key pro-fibrotic factors in LX-2 cells and mesenchymal stem cells (Ding et al., 2013; Beilfuss et al., 2015; Zhuang et al., 2019).

### 5.2 Alterations in vitamin D metabolism associated with biliary atresia

Serum 25(OH)D level is widely regarded as a reliable biomarker of vitamin D content in the human body (Jones, 2008). There are various methods of measurement, including solid-phase extraction from serum, radioimmunoassay using radioactive iodine tracers, and direct ultraviolet absorbance detection following separation by high-performance liquid chromatography (Hollis and Frank, 1985; Argao et al., 1992; Hollis, 1997). The normal range for serum 25(OH)D concentration in children is defined as above 50.0 nmol/L (Xiaoyang et al., 2010). Concentrations of vitamin D between 37.5 and 50.0 nmol/L are considered indicative of insufficiency, while concentrations equal to or less than 37.5 nmol/L indicate deficiency (Xiaoyang et al., 2010). Those equal to or less than 12.5 nmol/L represent severe deficiency (Xiaoyang et al., 2010). Accumulating epidemiological evidence suggests that Vitamin D deficiency is typically defined as a serum 25(OH)D concentration of less than 50 ng/ml (Hu et al., 2017).

Vitamin D deficiency is both common and severe in children with BA, a finding consistently supported by multiple clinical studies (Sun et al., 2021). As early as 1979, Japanese researchers documented the presence of 25(OH)D deficiency in children with BA (Kobayashi et al., 1979). In 1991, Tokita A et al. (Tokita et al., 1991) reported that 42.9% of children with BA before surgery presented with rickets. Among those with rickets, 16 cases exhibited severe vitamin D deficiency, with serum 1, 25(OH)_2_D levels of 6.14 ± 4.31 nmol/L, significantly lower than those of the control group (Tokita et al., 1991). Similarly, a study by Kobayashi A et al. (Kobayashi et al., 1974) found that 59% of infants with BA prior to surgery developed rickets secondary to severe vitamin D deficiency, with onset occurring between 1 and 4 months of age.

In recent years, a growing number of studies have confirmed the presence of 25(OH)D deficiency in children with BA. Ng J et al. (Ng et al., 2016) reported that 98.9% (91/92) of children with BA had 25(OH)D deficiency or insufficiency before LT, with only one child exhibiting normal 25(OH)D levels. Similarly, Zhuang P et al. (Zhuang et al., 2019) found that 96.3% (155/161) of children with BA presented with 25(OH)D deficiency or insufficiency before LT. In addition, two UK studies specifically focusing on infants with BA reported a high prevalence of vitamin D deficiency, ranging from 60.6% to 81% (Valta et al., 2008; Mancell et al., 2022). Meanwhile, several researchers have conducted in-depth investigations into the underlying causes of vitamin D deficiency in children with BA. One study excluded factors that could potentially cause poor vitamin D absorption or lead to vitamin D deficiency, such as preterm birth, physiological jaundice in infants, and progressive familial cholestasis (Song et al., 2025). It was found that the median serum 25(OH)D level in children with BA was only 8.05 nmol/L, significantly lower than the 47.67 nmol/L observed in children with non-BA cholestasis. This indicates that BA itself is an independent risk factor for low vitamin D levels. Sun S et al. (Sun et al., 2021) further demonstrated that children with BA not only had lower total vitamin D levels and significantly reduced 25(OH)D concentrations compared to controls, but also exhibited a markedly decreased 25(OH)D/vitamin D ratio. This finding reflects a significantly decreased rate of vitamin D activation in children with BA, pointing to both severe 25(OH)D deficiency and disrupted vitamin D activation. Additionally, studies have found that vitamin D deficiency in children with BA may result from impaired hepatic 25-hydroxylation due to reduced expression of CYP2R1 in the liver (Kimura et al., 1988; Sun et al., 2021). Deficiency of CYP2R1 impairs vitamin D activation, which may promote the proliferation and activation of HSCs and contribute to the progression of hepatic fibrosis in BA (Sun et al., 2021).

Vitamin D deficiency remains common in children with BA even at the stage of end-stage liver disease, after KPE, or following LT (Loeb et al., 2018). Battaglini G et al. (Battaglini et al., 1991) found that even in children with successful KPE, serum 25(OH)D levels remained lower than those in healthy controls in the absence of vitamin D supplementation. Notably, some of these children already exhibited signs of poor prognosis, such as cholangitis or cirrhosis (Battaglini et al., 1991). Despite undergoing LT, vitamin D insufficiency or deficiency persisted in 67% (133/199) of children with BA (Valta et al., 2008). Additionally, Teisseyre M et al. (Teisseyre et al., 2007) examined vitamin D status at multiple time points after LT in 20 children with BA aged 6 months to 2.4 years. Compared with pre-LT values, levels of 1, 25(OH)_2_D increased significantly three months after LT (Teisseyre et al., 2007). However, levels of 25(OH)D, a marker of vitamin D stores, did not show meaningful improvement (Teisseyre et al., 2007). No further increases in these parameters were observed during the subsequent nine months after LT (Teisseyre et al., 2007). Long-term follow-up data further revealed that among 40 children with BA followed for seven years after LT, 13% had 25(OH)D levels below 15 ng/mL (Tan et al., 2020). We hypothesize that the persistence of vitamin D deficiency in some children with BA after surgery may be related to impaired vitamin D metabolism or activation, as well as ongoing progression of hepatic fibrosis (Peng et al., 2019).

## 6 The positive feedback loop of the bile acid-gut microbiota-vitamin D axis in the initiation and progression of biliary atresia

**Figure 2:**
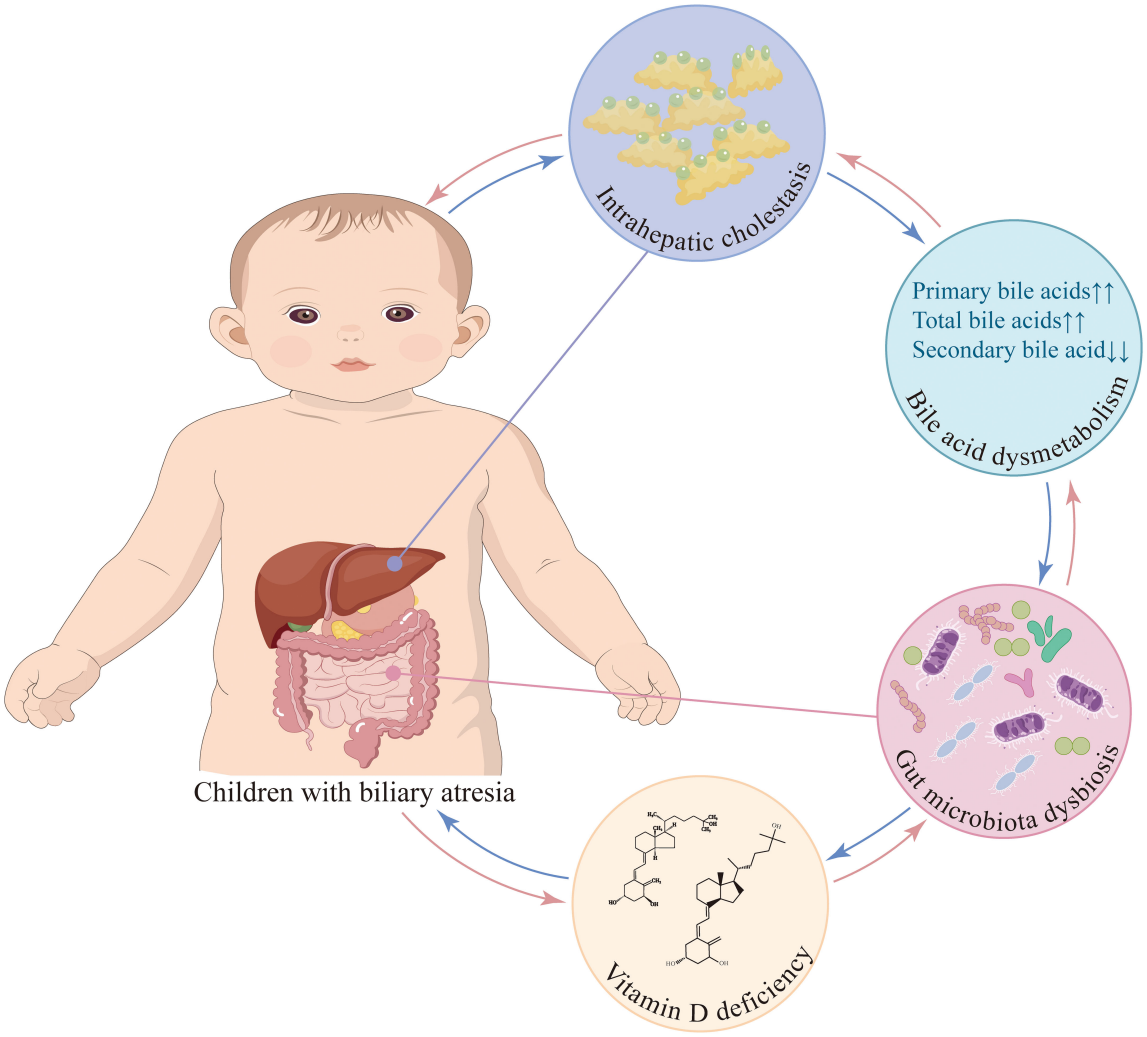
Schematic diagram of the bile acid-gut microbiota-vitamin D positive feedback loop in children with biliary atresia. Cholestasis-induced bile acid dysregulation promotes gut microbiota dysbiosis, which may impair vitamin D absorption and metabolism, forming a positive feedback mechanism (blue arrows). Vitamin D deficiency in turn exacerbates microbial imbalance and bile acid metabolic disorders, forming a negative feedback mechanism (pink arrows). Together, these interactions form a positive feedback vicious loop, a potential integrative framework, that may drive progressive liver injury and fibrosis in biliary atresia.

Multiple studies have demonstrated significant abnormalities in bile acid metabolism, gut microbiota composition and function, as well as vitamin D levels in children with biliary atresia. The positive feedback loop mechanism of the bile acid-gut microbiota-vitamin D axis is a potential integrative framework involved in the initiation and progression of biliary atresia. Exploring this axis may offer new insights into its pathogenesis and therapeutic strategies (**Figure 2**).

### 6.1 Positive feedback within the axis

In the positive feedback within the axis, disturbances in bile acid metabolism resulting from cholestasis may serve as a trigger for the subsequent cascade of pathological events.

#### 6.1.1 Biliary atresia-induced bile acid dysmetabolism

BA is an idiopathic neonatal cholestatic disorder characterized by abnormal narrowing, obstruction, or complete absence of the IHBDs and EHBDs (Averbukh and Wu, 2018). This leads to impaired bile acid excretion, impaired conversion of bile acids flowing from the biliary tract to the intestine for gut microbiota-mediated metabolism, as well as abnormalities in bile acid transport and synthesis (Song et al., 2021c; Wu et al., 2024). These disturbances result in intrahepatic cholestasis and bile acid dysmetabolism, leading to a disruption of bile acid homeostasis (Wu et al., 2024), which is characterized by marked increases in TBA and primary bile acids, along with a significant reduction in secondary bile acids. As shown in **Table 1**, Xinbei T et al. (Xinbei et al., 2021) also observed increased primary bile acids and TBA in bile duct ligation (BDL) mice, a widely used animal model for BA. In detail, Mingming L et al. (Mingming, 2020) found that GCA and the GCA/CDCA ratio increased in BA patients without treatment. Meanwhile, Song W et al. (Song et al., 2021a) reported that specific secondary bile acids, such as nor cholic acid (NORCA), allocholic acid (ACA), taurohyodeoxycholic acid (THDCA), and 3-dehydrocholic acid (3-DHCA), were decreased in BA patients without treatment.

The liver is a key organ for bile acid synthesis, transport, and metabolism (Li and Chiang, 2024). During cholestasis, the overload of bile acids in the liver plays a critical role in the pathogenesis of the disease. Shang T et al. (Shang et al., 2024) found that hepatic uptake transporters are down-regulated in cholestatic liver disease. Excess hydrophobic bile acids, such as GCDCA and TCDCA, are highly cytotoxic (Xinbei et al., 2021). They may induce apoptosis or necrosis of hepatocytes and cholangiocytes by triggering endoplasmic reticulum stress, mitochondrial dysfunction, reactive oxygen species (ROS) production, and disruption of primary cilia on cholangiocytes (Gong et al., 2016; Wang et al., 2022). These high concentrations of bile acids further activate pro-inflammatory pathways (Fickert and Wagner, 2017; Sutton et al., 2024). Furthermore, the hepatocyte injury and inflammation, in turn, can lead to abnormalities in bile acid transport, thereby contributing to bile acid dysmetabolism. In the early stage of BA, Yang H et al. (Yang et al., 2009) reported that inflammation leads to downregulation of bile acid transporters on the basolateral and canalicular hepatobiliary transporters. More specifically, Chen HL et al. (Chen et al., 2008) further found that in early BA, most canalicular and sinusoidal transporters are downregulated, including bile salt export pump (BSEP), sodium-dependent taurocholate cotransporting polypeptide (NTCP), and organic anion transporter (OATP). Moreover, human bile acid metabolism is subject to negative feedback regulation. Not only can elevated bile acids suppress CYP7A1 expression by activating the hepatic FXR-SHP axis and the intestinal FXR-FGF19-hepatic FGFR4/β-Klotho axis (Inagaki et al., 2005; Kim et al., 2007), but they can also engage the FXR/TGR5 signaling pathway to modulate bile acid metabolism (Wang et al., 2024). However, biliary obstruction in children with BA reduces bile flow to the intestine, leading to markedly decreased hepatic SHP expression, insufficient intestinal FXR activity or suppression of its downstream target FGF19 (Hasegawa et al., 2019; Yan et al., 2022). These disruptions impair the negative feedback inhibition of CYP7A1, thereby exacerbating bile acid synthesis and promoting hepatic fibrosis (Liu et al., 2020). Concurrently, reduced levels of secondary bile acids may attenuate activation of the FXR-TGR5-mediated anti-inflammatory signaling (Duboc et al., 2013; Sinha et al., 2020). In addition, accumulated toxic bile acids such as GCDCA promote hepatic fibrosis via activation of the NLRP3 inflammasome pathway (Hohenester et al., 2020; Feng et al., 2024b).

#### 6.1.2 Positive feedback of bile acid dysmetabolism on gut microbiota

Cholestasis-induced bile acid disturbances drive gut microbiota dysbiosis and intestinal barrier damage in children with BA. Specifically, bile acids exhibit both cytotoxic and antimicrobial properties (Baiocchi et al., 2019). Their antibacterial effects are mediated through multifactorial mechanisms, including membrane disruption, DNA damage, RNA structural alterations, and protein denaturation (Winston and Theriot, 2020). By shaping the composition and function of the gut microbiota, as well as influencing bacterial survival and growth, bile acids play a pivotal role in maintaining intestinal homeostasis (Thibaut and Bindels, 2022; Chen et al., 2023). On one hand, in children with BA, biliary obstruction leads to intrahepatic cholestasis and markedly reduced or absent bile flow to the intestine. This loss of bile acids diminishes their bacteriostatic effect in the gut, resulting in bacterial overgrowth, altered intestinal pH, and subsequent microbial dysbiosis characterized by enrichment of opportunistic pathogens and depletion of beneficial bacteria (Yajun, 2024; Xie et al., 2025). Yang et al. (Yang T. et al., 2022) further observed that *Bifidobacterium* abundance negatively correlates with conjugated bile acids such as GCA, TCA, TCDCA, and GCDCA, whereas *Rothia* negatively correlates with secondary bile acids including DCA and LCA. Beyond compositional shifts, multiple studies have reported functional alterations in the gut microbiota of BA. For example, sublethal concentrations of DCA, TCA, and taurodeoxycholic acid (TDCA) have been shown to disrupt bacterial nucleotide and carbohydrate metabolism in mice (Tian et al., 2020). On the other hand, Tilg H et al. (Tilg et al., 2022) demonstrated that cholestasis leads to dysregulation of gut-liver axis dynamics and impairs intestinal mucosal barrier function. Specifically, cholestasis reduces the abundance of BSH-producing beneficial bacteria, decreasing BSH activity and impairing bile acid deconjugation, thereby suppressing intestinal FXR/TGR5 and FXR-FGF19 signaling axes (Duboc et al., 2013; Li, 2019; Sinha et al., 2020). It also downregulates intestinal barrier proteins, including mucin-2, claudin-1(CLDN1), occludin (OCLD), and ZO-1 (Xie et al., 2025). These disruptions further alter gut microbiota composition, compromise intestinal barrier integrity, and increase intestinal permeability (Xu et al., 2023; Wang et al., 2024).

Meanwhile, bile acid dysmetabolism-induced gut microbiota dysbiosis and intestinal barrier damage may serve as potential contributors to disease progression in children with BA. Specifically, dysbiosis ignites intrahepatic inflammation (Chuang et al., 2001), and the increased intestinal permeability resulting from intestinal barrier damage facilitates translocation of pathogenic bacteria and endotoxins such as lipopolysaccharide (LPS) into the portal circulation, leading to bacterial translocation and endotoxemia, which further exacerbates liver injury and drives hepatic fibrogenesis (Wang et al., 2010; Gong et al., 2016). Song W et al. (Song et al., 2021a) found that *Klebsiella* spp. and *V. atypica* play important roles in the pathogenesis of BA by analyzing functional metabolism and clinical parameters. Moreover, they emphasized that liver injury in BA is directly or indirectly amplified by the intricate interplay among three interconnected factors, including disrupted bile acid metabolism, enrichment of *Klebsiella pneumoniae*, *V. atypica*, and *Enterococcus faecalis*, as well as the resulting disturbances in tryptophan metabolism (Song et al., 2021a). For instance, studies have shown that enriched *K. pneumoniae* can breach the epithelial barrier via virulence factors, capsular polysaccharide transport, and bacterial secretion systems, ultimately triggering bacterial translocation and hepatitis (Nakamoto et al., 2019; Patro and Rathinavelan, 2019).

Furthermore, after LT, restoration of the gut-liver axis has been observed in children with BA. Bile flow into the intestine lowers the intestinal pH, which inhibits the growth of pathogenic bacteria and helps maintain a balanced gut microbiota (Song et al., 2021c).

#### 6.1.3 Positive feedback of gut microbiota dysbiosis on vitamin D metabolism

Accumulating evidence suggests that alterations in the gut microbiota of children with BA may be associated with their vitamin D levels. A two-sample Mendelian randomization study found that vitamin D deficiency is associated with the genera *Allisonella*, *Eubacterium*, and *Tyzzerella* (Hou et al., 2025). A study in patients with obesity and metabolic syndrome on a Mediterranean diet found that decreased 25(OH)D is associated with reduced microbial diversity and alterations in *Bacteroides* and *Prevotella* (Boughanem et al., 2023). What’s more, a study in older men showed that circulating levels of the active vitamin D form 1, 25(OH)_2_D correlate with specific gut microbes such as *Blautia* and *Ruminococcus* (Thomas et al., 2020). Interestingly, some of these genera have also been observed to change in BA, as shown in **Tables 2**-**4**. Untreated BA children show reduced microbial diversity and decreased abundances of *Eubacterium*, *Bacteroides*, and *Blautia*. After KPE, *Bacteroides* abundance increased, whereas *Blautia* remained decreased. Following LT, *Blautia* and *Eubacterium* abundance increased compared with pre-LT values. Meanwhile, many studies observed that vitamin D deficiency is common in untreated BA children, and serum 1, 25(OH)_2_D levels increase significantly three months after LT (Teisseyre et al., 2007). These findings raise the possibility that vitamin D deficiency in children with BA may be causally linked to specific gut microbial features. However, direct evidence supporting this association in children with BA is currently lacking, and further studies are needed to validate it.

To understand how the gut microbiota might influence vitamin D levels, we examine potential mechanisms involving vitamin D absorption and metabolism. Tokita A et al. (Tokita et al., 1991) found that reduced serum 25(OH)D levels in preoperative BA patients are likely due to impaired intestinal vitamin D absorption. This may be associated with gut microbiota dysbiosis-induced intestinal barrier damage, leading to impaired absorption of 25(OH)D and consequently reduced circulating levels. Furthermore, using metagenomic sequencing, Wei S et al. (Wei et al., 2021) found that in 16 children with BA, gut microbiota diversity was significantly reduced before LT. After LT, abundances of *Roseburia*, *Eubacterium*, *Blautia*, *Clostridium*, and *Akkermansia* increased, accompanied by an upregulation of vitamin metabolism pathways (Wei et al., 2021). This observation suggests that gut microbiota dysbiosis may be associated with vitamin D metabolism and thus influence vitamin D levels in BA. More recently, Liu F et al. (Liu F. et al., 2024) further found that gut microbiota dysbiosis is involved in vitamin metabolism by analyzing functional pathways in children with BA. Studies have reported that beneficial bacteria capable of producing BSH are associated with the conversion of vitamin D to its active form or the enhancement of its bioavailability (Hou et al., 2025). As shown in **Tables 2**-**4**, children with BA without treatment exhibit gut microbiota dysbiosis, characterized by decreased abundances of BSH-producing beneficial bacteria such as *Clostridium*, *Bacteroides*, *Enterococcus*, and *Eubacterium*, which may affect vitamin D metabolism and bioavailability.

Interestingly, Bora SA et al. (Bora et al., 2018) found that the gut microbiota can regulate vitamin D metabolism through FGF23. FGF23 is a hormone primarily secreted by osteocytes and osteoblasts and acts as a potent regulator and a key mediator of vitamin D metabolism that is influenced by the gut microbiota (Shimada et al., 2004; Bora et al., 2018). It regulates phosphate and vitamin D metabolism by binding to its receptor FGFR and its co-receptor Klotho (Bora et al., 2018). In germ-free mice, reduced 1, 25(OH)_2_D levels were attributed to increased FGF23 levels (Bora et al., 2018). Increased FGF23 inhibits parathyroid hormone (PTH) secretion and induces CYP24A1 expression, resulting in decreased production of 1, 25(OH)_2_D (Shimada et al., 2004). This condition was reversed upon colonization with commensal bacteria. Specifically, after introduction of microbiota into germ-free mice via colon contents or fecal transplantation, the microbiota may suppress FGF23 by inducing inflammatory responses, leading to a significant increase in serum 1, 25(OH)_2_D levels (Bora et al., 2018). However, whether gut microbiota dysbiosis contributes to vitamin D deficiency through specific bacterial taxa, FGF23, or related pathways in BA remains to be investigated.

Moreover, some studies have provided additional insights that vitamin D may be an important regulator of liver fibrosis in children with BA. Using a BDL animal model, a widely used model of BA, Sun et al. (2021) found that administration of exogenous 1, 25(OH)_2_D reduced the expression of fibrosis-related genes from BA patients *in vitro*. Both *in vitro* and *in vivo* experiments indicated that vitamin D not only suppresses pro-fibrotic genes such as Col-1α1 and TIMP-1 but also promotes the anti-fibrotic gene MMP9 (Sun et al., 2021), suggesting that deficiency of active vitamin D may promote hepatic fibrogenesis. In clinical studies, Zhuang et al. (2019) reported a negative correlation between serum 25(OH)D levels and the degree of liver fibrosis in 161 children with BA. Similarly, Peng et al. (2019) found an inverse association between 25(OH)D levels and liver shear wave elastography in 33 BA children after KPE. However, the precise mechanisms underlying the relationship between vitamin D deficiency and liver fibrosis in BA, as well as the impact of the severity of deficiency on liver pathology, remain unexplored and warrant further investigation.

Taken together, the positive feedback within the axis may orchestrate a potential pathological cascade in children with BA, initiated by cholestasis, followed by gut microbiota dysbiosis and culminating in vitamin D deficiency, all of which may collectively drive the progression of liver injury and fibrosis.

### 6.2 Negative feedback within the axis

The negative feedback within the axis may play a potential role in the progression of BA.

#### 6.2.1 Negative feedback of vitamin D deficiency on gut microbiota

The interaction between gut microbiota and vitamin D is not unidirectional but rather a dynamic bidirectional relationship. Gut microbiota dysbiosis not only reduces 1, 25(OH)_2_D production but also downregulates VDR expression, compromising vitamin D activation and signaling (Thomas et al., 2020). Conversely, vitamin D deficiency and impaired VDR signaling in children with BA may exert negative feedback on the composition and function of the gut microbiota, further exacerbating its dysbiosis.

Studies have shown that human VDR is a key host factor shaping the gut microbiota (Wang et al., 2016). Vitamin D can influence gut microbiota homeostasis by activating VDR signaling in intestinal bacteria (Zhang et al., 2019). Together, vitamin D and its receptor VDR promote gut microbiota homeostasis by maintaining intestinal barrier function, exerting antimicrobial and anti-inflammatory effects, and enhancing immune tolerance to gut microbiota, thereby regulating the composition and function of the microbial community (Jin et al., 2015; Ogbu et al., 2020). Vitamin D helps maintain intestinal mucosal barrier homeostasis by increasing the expression of tight junction proteins and reducing intestinal epithelial cell apoptosis (Del Pinto et al., 2017). Similarly, VDR regulates the expression of the tight junction proteins zonulin occluden-1, zonulin occluden-2 by upregulating claudin-2 and claudin-12 and downregulating adherin-17 in the intestine (Zhang et al., 2013). In addition, both 1, 25(OH)_2_D and VDR induce macrophages to produce antimicrobial peptides such as β-defensins and cathelicidin, which selectively kill pathogenic bacteria to maintain a healthy gut microbial ecosystem (Wang et al., 2004; Gombart et al., 2005; Hewison, 2011). More importantly, vitamin D and VDR not only regulate the innate immune response to the microbiome (Cantorna et al., 2014), but also enhance immune tolerance to gut microbiota by mediating host immune-microbe interactions (Gubatan et al., 2026). Specifically, vitamin D/VDR signaling may increase IgA-bound beneficial bacteria with the capacity to produce SCFAs and secondary bile acids and decrease IgG-bound proinflammatory bacteria through induction of IgA+ B cells via increased B cell activating factor (BAFF) signaling between plasmacytoid dendritic cells (pDCs) and B cells, and through induction of anti-inflammatory α4β7+ B and T regulatory cells, collectively boosting immune tolerance to the gut microbiota (Gubatan et al., 2026). Moreover, vitamin D may exert additional anti-inflammatory effects through the selection of IgA-bound gut bacteria that produce SCFAs and secondary bile acids (Gubatan et al., 2026).

Synthesizing evidence from both animal studies and human trials, this section examines how vitamin D deficiency and impaired VDR signaling may shape the gut microbiota.

Multiple studies have demonstrated that deficiencies in vitamin D or its metabolites, 25(OH)D or 1, 25(OH)_2_D, can disrupt gut microbial homeostasis. In an infectious model of colitis, vitamin D deficiency has been shown to alter fecal microbiota composition by impairing intestinal epithelial barrier function and promoting gut inflammation (Assa et al., 2014). Zhu W et al. (Zhu et al., 2019) reported that loss of 1, 25(OH)_2_D in CYP27B1 knockout mice leads to disruption of the colonic mucus barrier, subsequently triggering gut microbiota dysbiosis. Similarly, Assa A et al. (Assa et al., 2014; Assa et al., 2015) found that vitamin D-deficient mice were not only more susceptible to intestinal epithelial barrier dysfunction and increased intestinal permeability, but also exhibited marked shifts in gut microbiota composition, including an increased abundance of *Actinobacteria*, *Proteobacteria*, and *Bacteroidetes* phyla. As shown in **Table 2**, Yan M et al. (Yan et al., 2022) also found that *Bacteroidetes* increased in a BDL rat model, and Xu X et al. (Xu et al., 2023) reported that *Proteobacteria* are increased in children with BA without treatment. A study in vitamin D-deficient children further revealed that these children exhibit gut microbiota dysbiosis, characterized by reduced microbial diversity and a lower abundance of beneficial genera such as *Bifidobacterium* and *Akkermansia* (Singh et al., 2022). Also, as shown in **Table 2**, many studies have found that *Bifidobacterium* and *Akkermansia* are decreased in children with BA without treatment. These raise the possibility that vitamin D deficiency in children with BA may contribute to gut barrier dysfunction and subsequent gut microbiota dysbiosis.

Interestingly, vitamin D deficiency is often accompanied by VDR gene mutations, which also cause gut microbiota dysbiosis and change its functional profile (Jin et al., 2015; Huang et al., 2019). Intestinal VDR deficiency increases bacterial load and contributes to gut microbiota dysbiosis, characterized by a marked reduction in butyrate-producing bacteria such as *Parabacteroides* and *Lactobacillus*, while *Clostridium* and *Bacteroides* become enriched (Ooi et al., 2013; Wang et al., 2016; Bakke and Sun, 2018; Sukik et al., 2023). Gut microbiota dysbiosis can impair intestinal barrier function and increase intestinal permeability, which facilitate the translocation of bacterial products such as lipopolysaccharide into the portal circulation (Jee et al., 2017). These microbe-associated molecular patterns activate hepatic immune cells such as Kupffer cells, and exacerbate liver inflammation, oxidative stress, and fibrosis via TLR4/NF-κB pathways (Jee et al., 2017). Based on these findings, it is plausible that the interplay among vitamin D deficiency, impaired VDR signaling, and gut microbiota dysbiosis may drive further progression of BA.

However, most existing studies on vitamin D-induced gut microbiota alterations have focused on diseases such as diabetes or colitis. Direct experimental evidence supporting the specific mechanisms linking vitamin D deficiency to gut microbiota changes in the context of BA is currently lacking, and further investigation is needed.

#### 6.2.2 Negative feedback of gut microbiota dysbiosis on bile acid metabolism

Moreover, gut microbiota dysbiosis can exert negative feedback on bile acid homeostasis by influencing the enzymes and pathways involved in bile acid metabolism (Zhang et al., 2022).

The gut microbiota produces key enzymes involved in converting primary bile acids into secondary bile acids to maintain bile acid homeostasis, including BSH and 7α-dehydroxylase (Zhang et al., 2025). BSH hydrolyses the amino-acid moiety of conjugated primary bile acids. This enzymatic activity is predominantly found in bacterial phyla such as *Bacteroidetes* phylum, *Firmicutes* phylum, and *Actinomycetota* phylum, as well as in genera including *Lactobacillus*, *Bacteroides*, *Bifidobacterium*, *Clostridium*, *Listeria*, and *Enterococcus* (Cai et al., 2022; Collins et al., 2023). 7α-dehydroxylase, produced by gut microbes such as *Clostridium* and members of the *Firmicutes*, catalyzes the oxidation, isomerization, and dehydroxylation of bile acids (Jia et al., 2018; Guzior and Quinn, 2021). Anaerobic bacteria in the small intestine metabolize the conjugated primary bile acids CA and CDCA into the secondary bile acids DCA and LCA via 7α-dehydroxylation (Masubuchi et al., 2016). In contrast, as shown in **Table 2**, many studies found that in children with BA, gut microbiota dysbiosis is accompanied by a marked reduction in bacteria that express BSH or 7α-dehydroxylase, such as *Bifidobacterium*, *Lactobacillus*, and members of the *Firmicutes*. This decline reduces the activity of microbial enzymes involved in host bile acid metabolism, leading to impaired secondary bile acid production (Cai et al., 2022; Collins et al., 2023). As shown in **Table 1**, many studies have found that secondary bile acids are decreased in children with BA without treatment. Consequently, the secondary bile acid pool becomes smaller, thereby further aggravating bile acid metabolism disorder (Vrieze et al., 2014).

In addition to modulating enzymes involved in intestinal bile acid metabolism, the gut microbiota can also influence the expression of key hepatic enzymes, such as CYP7A1, CYP7B1, CYP8B1, and CYP27A1, by interfering with the FXR-FGF19 negative feedback pathway (Cai et al., 2022). Bile acids serve as signaling messengers between the liver and the gut (Zhang et al., 2022). Li F et al. (Li et al., 2013) found that a reduction in secondary bile acids inhibits intestinal FXR signaling, leading to increased hepatic bile acid synthesis via upregulation of CYP7A1, the rate-limiting enzyme in bile acid synthesis, thereby worsening bile acid metabolism disorder and cholestasis. Meanwhile, gut microbiota dysbiosis not only diminishes secondary bile acid production but also reduces BSH activity owing to a decline in *Lactobacillus*, then leading to elevated levels of the conjugated primary bile acid tauro-β-muricholic acid (T-β-MCA) (Jiang et al., 2015; Yan et al., 2022). Liu Y et al. (Liu et al., 2020) demonstrated that T-β-MCA levels were significantly increased in BDL mice. In healthy individuals, T-β-MCA acts as an FXR antagonist. Intestinal bacterial BSH deconjugates T-β-MCA, lowering its levels in order to activate the intestinal FXR signaling pathway, which in turn suppresses CYP7A1 expression and bile acid synthesis to maintain bile acid homeostasis (Joyce et al., 2014). Based on this, elevated T-β-MCA suppresses the intestinal FXR signaling pathway and reduces FGF19 levels, leading to increased hepatic CYP7A1 transcription, enhanced bile acid synthesis, and an expanded bile acid pool (Xie et al., 2017). These changes may disrupt bile acid homeostasis and further aggravate intrahepatic cholestasis (Xie et al., 2017). Moreover, *Clostridium* and *Bacteroides* of the *Firmicutes* phylum are believed to regulate bile acid synthesis through the FXR-FGF19 negative feedback axis (Chiang, 2015). As shown in **Table 2**, many studies found a reduction in *Clostridium* and *Bacteroides* in children with BA. This reduction may impair this negative feedback mechanism, further exacerbating bile acid metabolism disorder and cholestasis. These processes may further promote intrahepatic inflammation, liver injury, and hepatic fibrosis in children with BA.

Taken together, the negative feedback within the axis may further aggravate gut microbiota dysbiosis, bile acid metabolism disorder, and cholestasis, thereby potentially driving adverse disease progression in patients with BA.

In summary, the positive feedback loop of the bile acid-gut microbiota-vitamin D axis may establish a pathogenic vicious cycle in the initiation and progression of BA, centered on bile acid metabolism disorder, gut microbiota dysbiosis, and vitamin D deficiency. However, experimental evidence specifically addressing the interconnections and mechanistic details among these three components in the context of BA is still lacking. Relevant studies rely primarily on selected human cohorts or mouse models, needing large-scale population-based studies to further clarify the underlying molecular mechanisms.

## 7 Exploring new strategies for diagnosis and treatment of biliary atresia based on the bile acid-gut microbiota-vitamin D axis

In recent years, the concept and significance of the bile acid-gut microbiota-vitamin D axis has been investigated in relation to various diseases. Li A et al. (Li A. et al., 2023) proposed that this axis plays a key role in determining oocyte quality and embryonic development. Lin HR et al. (Lin et al., 2023) proposed that circulating 25(OH)D and the gut microbiota-bile acid axis play critical roles in human metabolic health, and that this axis mediates the association between plasma 25(OH)D and metabolic syndrome. The same study also found that four gut microbial genera, 11 faecal bile acid species and circulating vitamin D were associated with the risk of multiple sclerosis, highlighting the significant mediation of the link between vitamin D and multiple sclerosis by the gut microbiota-bile acid axis (Lin et al., 2023). However, no definitive preoperative diagnostic test for BA is currently available in clinical practice, and establishing an optimal early diagnostic strategy remains challenging (Song et al., 2025). There is an urgent clinical need to identify non-invasive indicators or novel biomarkers for the early diagnosis of BA in children, in order to improve the timeliness and accuracy of early diagnosis (Sun et al., 2022). Therefore, investigating the potential role of the positive feedback loop of the bile acid-gut microbiota-vitamin D axis in the initiation and progression of BA may deepen our understanding of its pathogenesis and provide new insights for research on its diagnosis and treatment.

### 7.1 Bile acids as a novel approach in the diagnosis and treatment of biliary atresia

The disruption of bile acid metabolism caused by BA contributes to the progression of the disease towards liver damage and liver fibrosis. This not only provides a new perspective for understanding the pathological mechanisms, but also offers a novel approach to the development of new diagnostic markers and therapeutic targets.

#### 7.1.1 Characteristic bile acid profile as a novel biomarker for biliary atresia

In terms of diagnosis, the characteristic bile acid profile in BA shows great potential as a non-invasive biomarker. Zhou et al. (2015) demonstrated that children with BA exhibit a plasma bile acid profile that is significantly different from those with neonatal hepatitis syndrome (NHS) or who are healthy controls. This profile is characterised by elevated TCDCA, reduced CDCA and a markedly increased TCDCA/CDCA ratio (Zhou et al., 2015). This ratio achieved an area under the receiver operating characteristic curve (AUC) of 0.923 (95% CI: 0.862-0.984) for distinguishing BA from NHS, indicating excellent diagnostic performance (Zhou et al., 2015). Furthermore, Mingming L’s study found that GCA levels and the GCA/CDCA ratio within the characteristic serum bile acid profile of children with BA have diagnostic value in distinguishing BA from other non-BA biliary obstructive disorders (Mingming, 2020). Preoperative serum GCA levels greater than 21μmol/L or a GCA/CDCA ratio greater than 846 may serve as important supportive evidence for the diagnosis of BA (Mingming, 2020). Concurrently, using more advanced mass spectrometry, Obatake M et al. (Obatake et al., 2002) found that serum GCDCA concentration can differentiate BA from non-BA cholestatic diseases, with a sensitivity of 100% and a specificity of 83.3%, opening new avenues for precise diagnosis.

#### 7.1.2 Bile acid-targeted therapies based on the positive feedback loop in biliary atresia

Based on in−depth research into the bile acid-gut microbiota-vitamin D axis in children with biliary atresia, therapies targeting bile acids can not only regulate metabolic homeostasis, but also modulate the gut microbiota and vitamin D levels. Currently, the main therapeutic options for treating disorders of bile acid metabolism in children with BA include various bile acids, bile acid analogues, and receptor agonists.

Ursodeoxycholic acid (UDCA) is a potent bile acid that exhibits both choleretic and cholangioprotective properties (Trampert and Beuers, 2024). It is commonly used to treat neonatal cholestasis and is also an FDA-approved first-line treatment for biliary liver disease (Paumgartner and Beuers, 2004; Lynch et al., 2023). Not only does UDCA regulate bile acid homeostasis by markedly increasing faecal UDCA and TBA levels in neonates with cholestasis, it also modulates gut microbiota composition, leading to an enrichment of BSH-harbouring *Clostridium perfringens* (Lynch et al., 2023). What’s more, postoperative UDCA treatment following KPE has been associated with improved clinical outcomes in children with BA (Yang T. et al., 2022). Alongside UDCA, ileal bile acid transporter (IBAT) inhibitors such as maralixibat (NCT04524390) and odevixibat (NCT04336722) can help to restore bile acid homeostasis by reducing bile acid reuptake in the distal ileum (Jeyaraj et al., 2024). These inhibitors may also limit bile acid accumulation in children with BA after KPE (Jeyaraj et al., 2024).

Within the positive feedback loop of the bile acid-gut microbiota-vitamin D axis in children with BA, hepatic synthesis of FXR, SHP, and FGF19 is markedly reduced, while intestinal FXR signaling is suppressed and the FXR-TGR5 pathway is damaged (Hasegawa et al., 2019; Sinha et al., 2020; Yan et al., 2022). Consequently, modulating receptors involved in bile acid metabolism has emerged as an interesting therapeutic strategy. In particular, targeting FXR activity plays an important role in regulating bile acid synthesis and maintaining gut microbial homeostasis in children with BA. Currently available FXR agonists include GW4064, obeticholic acid (NCT05321524), turofexorate isopropyl (WAY-362450), and cilofexor (GS-96740) (Trauner et al., 2019; Schwabl et al., 2021; Feng et al., 2024b). Administration of GW4064 counteracts the antagonistic effect of T-β-MCA on FXR and substantially improves gut microbiota composition at the phylum level (Song et al., 2023; Xie et al., 2025). Obeticholic acid (OCA), a modified primary bile acid, is a potent FXR agonist. Its biological activity is dozens of times greater than that of CDCA, making it the most promising FXR-targeting drug for the treatment of BA (van Golen et al., 2018). Not only does OCA induce increased levels of the FXR target gene SHP in the liver of the rats subjected to reversible bile duct ligation (rBDL), it also activates the intestinal FXR signaling pathway, thereby increasing FGF19 production (van Golen et al., 2018). This leads to suppression of CYP7A1, thereby reducing bile acid synthesis and alleviating cholestasis in BA. Meanwhile, OCA markedly inhibits apoptosis of intestinal epithelial cells, thereby alleviating gut barrier dysfunction (Yan et al., 2022). It also increases the abundance of beneficial bacteria and decreases the abundance of harmful bacteria, improving the composition and metabolic function of the dysbiotic gut microbiota and further helping to restore microbial homeostasis in BDL rats (Yan et al., 2022). In addition, the improvement in cholestasis, correction of bile acid metabolism disorders and restoration of gut microbiota dysbiosis all promote intestinal absorption of vitamin D, thereby raising vitamin D levels in children with BA. However, this speculation currently lacks direct experimental evidence in children with BA. The role of intestinal FXR in regulating the intestinal mucosal barrier and gut microbiota homeostasis remains to be investigated further.

FXR agonists not only modulate bile acid metabolism and the gut microbiota, but also hold promise as potential therapeutic agents for liver fibrosis and cirrhosis (Kim et al., 2016). Emerging evidence suggests that FXR activation may suppress TGF-β/SMAD3 signaling through the FXR-SHP regulatory cascade, leading to the suppression of HSC proliferation and thereby attenuating the progression of liver fibrosis and cirrhosis (Renga et al., 2011; Feng et al., 2024b). Additionally, BAR502 is a dual agonist of FXR and TGR5, which suppresses bile acid synthesis by upregulating the expression of SHP and FGF19, resulting in the reversal of liver fibrosis in mouse models of liver fibrosis induced by carbon tetrachloride (CCl_4_) (Carino et al., 2017). Furthermore, FGF19 and its analogues, including NGM282 and M70, have been extensively studied as a novel therapeutic approach and possess potent anti-cholestatic and anti-fibrotic activity (Zhou et al., 2017; Harrison et al., 2020). In a randomized trial involving healthy human volunteers, Luo J et al. (Luo et al., 2014) found that NGM282 may exert beneficial effects in the treatment of cholestasis. In a mouse model of obstructive extrahepatic and intrahepatic cholestasis, NGM282 suppressed hepatic CYP7A1 expression, thereby reducing hepatic bile acid synthesis and serum liver enzyme levels, thus preventing cholestatic liver injury (Zhou et al., 2016; Yu et al., 2023).

Consequently, modulating bile acid metabolism through the use of drugs such as UDCA, FXR agonists, FGF19 and its analogues represents a novel and viable therapeutic approach for potentially preventing and alleviating bile stasis, gut dysbiosis, vitamin D deficiency and liver fibrosis in children with BA.

### 7.2 Potential diagnostic value and therapeutic benefits of the gut microbiota in biliary atresia

As a critical component of the bile acid-gut microbiota-vitamin D axis, the gut microbiota has garnered increasing attention for its involvement in the initiation and progression of biliary atresia. This not only offers a new perspective on revealing its pathogenesis, but also highlights its potential value as a non-invasive diagnostic marker and a promising therapeutic target.

#### 7.2.1 Altered gut microbiota as a potential diagnostic marker for biliary atresia

The characterisation of the gut microbiota in children with BA differs significantly from that in healthy controls and individuals with non-BA cholestatic diseases. These differences in gut microbiota could potentially be used as non-invasive biomarkers for diagnosing and predicting the prognosis of BA (Xu et al., 2023). Song et al. (2021a) characterized the gut microbiota across different stages of BA and found that *Klebsiella*, *Streptococcus*, *Veillonella*, and *Enterococcus* were consistently predominant, suggesting their potential use as diagnostic biomarkers for BA. Sun X et al. (Sun et al., 2022) reported that *Enterococcus*, *Ralstonia*, and other genera may serve as biomarkers to differentiate BA from non-BA cholestatic liver diseases. Consistent with this, Yajun L et al. (Yajun, 2024) identified *Enterococcus*, *Staphylococcus*, and *Bifidobacterium longum* as candidate biomarkers for distinguishing between children with BA and healthy infants.

Meanwhile, the abundance ratio of the gut microbiota has potential diagnostic value in BA. Both Xu X et al. (Xu et al., 2023) and Wang J et al. (Wang et al., 2020) identified the *Streptococcus*/*Bacteroides* ratio as a diagnostic biomarker for children with BA. Using databases and clinical data, Xu X et al. (Xu et al., 2023) further proposed that the *Streptococcus*/*Eggerthella* ratio is the optimal microbial marker for distinguishing between BA and non-BA cholestatic diseases. Liu et al. (Liu F. et al., 2024) demonstrated that the *Klebsiella*/*Bifidobacterium* ratio was significantly different in children with BA compared to healthy controls, which holds promise as a non-invasive diagnostic biomarker.

Furthermore, van Wessel et al. (2021) noted that the composition of the gut microbiota in children with BA before KPE is closely linked to the clearance of jaundice, which is a critical predictor of long-term outcomes. Recent studies have also shown that the abundance of *Bifidobacterium* is negatively correlated with cholestasis and could be used as an indicator of successful KPE in children with BA (Guo et al., 2018; Wang et al., 2019; Li et al., 2022).

#### 7.2.2 Potential therapeutic benefits of gut microbiota modulation based on the positive feedback loop

The gut microbiota not only serves as a non-invasive diagnostic marker for children with BA, but is also a potentially modifiable risk factor in BA, offering potential therapeutic targets for these children (Jain et al., 2021).

Based on the positive feedback loop of the bile acid-gut microbiota-vitamin D axis, interventions that target the gut microbiota may concurrently influence gut microbiota composition, bile acid metabolism, and vitamin D levels. Children with BA display characteristic alterations in the gut microbiota, marked by a reduction in butyrate-producing beneficial bacteria and an enrichment of opportunistic pathogens. Modulating the gut microbiota therefore may offer a promising avenue for developing novel therapies in children with BA. In recent years, several therapeutic drugs targeting gut microbiota dysbiosis have been developed, including probiotics, prebiotics, synbiotics, and fecal microbiota transplantation (FMT) (Song et al., 2021c; Collins et al., 2023).

Among probiotics, BSH-containing *Bifidobacterium* and *Lactobacillus* are the most commonly used (Tanaka et al., 1999). By regulating gut microbiota composition, they help maintain microbial homeostasis and restore intestinal mucosal barrier integrity via modulation of tight junction expression in intestinal epithelial cells (Yoon and Sun, 2011; Xiong et al., 2021; Yang KM. et al., 2021; Laterza et al., 2025). Probiotics also facilitate the deconjugation, dehydrogenation, dehydroxylation, and isomerization of primary bile acids in the ileum, generating hydrophobic bile acids and increasing fecal bile acid excretion (Wahlström et al., 2016; Fuchs and Trauner, 2022). They also activate the intestinal FXR-FGF19 pathway and downregulate ASBT expression, reducing hepatic bile acid synthesis and intestinal bile acid reabsorption, thereby ameliorating cholestasis and maintaining bile acid homeostasis (Degirolamo et al., 2014; Martoni et al., 2015; Yu et al., 2023). Moreover, they exert anti-inflammatory and anti-fibrotic effects in the liver (Lu et al., 2025). Consequently, these effects alleviate cholestatic liver injury, prevent hepatic fibrosis, and promote recovery of liver function (Liu et al., 2020; Wu et al., 2024). For example, *Lactobacillus rhamnosus GG* (LGG) is a probiotic. Liu Y et al. (Liu et al., 2020) treated BDL mice with LGG and found that it altered the composition of the gut microbiota and increased the abundance of gut bacteria with BSH activity, such as those belonging to the phyla *Firmicutes* and *Actinobacteria*. Meanwhile, it enhanced the intestinal FXR-FGF15 signaling pathway, leading to suppression of bile acid synthesis and promotion of bile acid excretion, resulting in significantly reduced hepatic bile acid levels (Liu et al., 2020). These changes alleviated liver inflammation, injury, and fibrosis in BDL mice (Liu et al., 2020). In addition, probiotics can increase the abundance of beneficial bacteria by producing beneficial metabolites, such as SCFAs, thereby improving bile acid metabolism (Yoon and Sun, 2011). Jee JJ et al. (Jee et al., 2022) showed that butyrate administration to pregnant mice conferred resistance to experimental BA in their offspring, which was associated with intestinal enrichment of *Bacteroidetes* and *Clostridia*.

Prebiotics are dietary supplements or fibres that are not digested by the host, but which selectively stimulate the growth and activity of beneficial gut bacteria (Zheng et al., 2019). Examples include fructooligosaccharides (FOS), inulin and galactooligosaccharides (GOS) (Zheng et al., 2019). When fermented by the gut microbiota, prebiotics generate SCFAs, particularly butyrate. These not only improve gut health and restore microbial balance, but also indirectly enhance bile acid metabolism (Rovinaru and Pasarin, 2020). Furthermore, faecal microbiota transplantation (FMT) has been employed in the treatment of patients with gut microbiota dysbiosis and bile acid metabolism disorders (Liang et al., 2020; Wu et al., 2024). Alongside the beneficial effects of probiotics and FMT administered before surgery, early supplementation with probiotics or prebiotics in children with BA following KPE may help to restore the gut microbiota, prevent postoperative cholangitis and improve clinical outcomes (Zheng et al., 2022; Jain et al., 2024). Furthermore, probiotic supplementation following LT in children with BA is a worth-considering clinical treatment strategy.

Based on the positive feedback loop, in addition to their role in improving gut microbiota dysbiosis and disorders of bile acid metabolism, probiotics and prebiotics may also help to correct vitamin D deficiency in children with BA. Probiotic strains such as LGG and Lactobacillus plantarum (LP) have been shown to increase vitamin D levels and enhance VDR expression and activity (Shang and Sun, 2017). Jones ML et al. (Jones et al., 2013) reported that the oral administration of the BSH-active probiotic strain Lactobacillus reuteri (NCIMB 30242) increased circulating 25(OH)D levels. Additionally, Gokhale S et al. (Gokhale and Bhaduri, 2019) discovered that prebiotic supplementation with FOS or inulin may alleviate vitamin D deficiency by modulating the composition of the gut microbiota and promoting vitamin D biosynthesis. Abboud M et al. (Abboud et al., 2020) observed that probiotics enhance vitamin D absorption in a dose-dependent manner, but the precise mechanisms remain to be elucidated.

These findings highlight the therapeutic benefits of gut microbiota in BA. Combined probiotic and prebiotic use may provide further benefits. Probiotic administration modulates the gut microbiota and is generally well tolerated, with only mild side effects such as flatulence or abdominal bloating, which supports its clinical applicability (Shang and Sun, 2017; Cejun et al., 2022). Although animal experiments and human studies have shown that gut microbiota-targeted interventions can improve gut dysbiosis and bile acid dysmetabolism, direct evidence for their ability to correct vitamin D deficiency in BA is still lacking, and the underlying correlations and mechanisms remain to be explored. Besides, existing studies on probiotic applications are generalised and non-specific (Shang and Sun, 2017). Furthermore, the clinical efficacy and safety of probiotics in patients have long been a subject of controversy (Yu et al., 2023). Consequently, there is a need to develop personalised gut microbiota treatment therapies based on the characteristic changes in the gut microbiota of children with BA. It is also essential to conduct long-term clinical trials to further investigate the specific clinical efficacy and safety of gut microbiota modulation in alleviating symptoms of children with BA.

### 7.3 Vitamin D as a simple diagnostic marker and therapeutic target in biliary atresia

Vitamin D is a key component of the bile acid-gut microbiota-vitamin D axis. It is frequently deficient in children with BA, and it has the potential to be used as a simple diagnostic marker and therapeutic target for the disease.

#### 7.3.1 25(OH)D as a simple diagnostic marker for biliary atresia

Zuo K et al. (Zuo et al., 2019) proposed that changes in vitamin D_3_ levels, the precursor of vitamin D, could be used as biomarkers to diagnose and monitor various clinical diseases. In a recent study, Song F et al. (Song et al., 2025) reported that 25(OH)D levels showed markedly improved diagnostic performance in distinguishing BA from other cholestatic diseases, achieving a sensitivity of 95%, a specificity of 96.08%, a positive predictive value of 95%, a negative predictive value of 96.08%, and an accuracy of 95.6%. They also suggested that the 25(OH)D level is a simple yet underutilised novel diagnostic approach that serves as a useful biomarker for BA (Song et al., 2025). In particular, preoperative serum 25(OH)D deficiency has been identified as a potential biomarker for early lung infection following LT in children (Tan et al., 2020).

#### 7.3.2 Potential therapeutic target of vitamin D supplementation based on the positive feedback loop

Vitamin D is a potential therapeutic target for children with BA. Presently, supplementation strategies primarily rely on the oral administration of vitamin D supplements, which can correct vitamin D deficiency to some extent. In a study by Daum et al. (1976), three children with extrahepatic BA received 50 μg of 25(OH)D daily, resulting in increased serum 25(OH)D levels and improved rickets symptoms. Further, a randomised clinical trial suggested that vitamin D supplementation may contribute to preventing and treating liver fibrosis, as well as improving liver function (Komolmit et al., 2017; Ebrahimpour-Koujan et al., 2019; Izadi et al., 2020). Nevertheless, it should be noted that LT alone does not sufficiently correct vitamin D deficiency, and additional oral vitamin D supplementation remains necessary (Veraldi et al., 2020). However, evidence from studies on the efficacy of vitamin D supplementation in children with BA who have vitamin D deficiency is currently lacking.

Moreover, based on the positive feedback loop, vitamin D supplementation may have the potential to exert therapeutic effects in children with BA by modulating the gut microbiota, thereby influencing intestinal microecology. Studies have shown that vitamin D supplementation not only corrects vitamin D deficiency, but also positively influences gut microbiota composition (Hussein et al., 2022). It increases microbial diversity, promotes the abundance of beneficial bacteria such as *Bifidobacterium* and *Akkermansia*, along with significantly reduces opportunistic pathogens (Bashir et al., 2016; Singh et al., 2020; Sukik et al., 2023). At the same time, Mandle HB et al. (Mandle et al., 2019) reported that vitamin D supplementation significantly upregulated the expression of tight junction proteins, including CLDN1, OCLD, and mucin-12 (MUC12), thereby improving impaired intestinal barrier function. Furthermore, by ameliorating gut microbiota dysbiosis and impaired intestinal barrier, vitamin D supplementation may help maintain bile acid homeostasis.

However, it is interesting to note that excessive vitamin D intake can have adverse effects on patients, such as shifting the microbial composition toward a more inflammatory faecal microbiome (Ghaly et al., 2018). Therefore, the supplementation of vitamin D should take into account appropriate doses and concentrations, and it is necessary to tailor vitamin D dosages to the individual (Aggeletopoulou et al., 2023; Hou et al., 2025). In addition, Argao EA et al. (Argao et al., 1992) studied 8 children with severe chronic cholestasis, including 3 with extrahepatic BA, and found that when vitamin D supplements alone are insufficient, combining them with d-α-tocopheryl polyethylene glycol 1000 succinate (TPGS) may enhance absorption in patients who have a deficiency of bile acids in the intestinal lumen, thereby gradually restoring serum 25(OH)D concentrations to normal levels. Meanwhile, a randomized controlled trial found that cyclodextrin formulations can also strengthen vitamin D_3_ supplementation (Nowak et al., 2021).

Although growing evidence suggests that oral administration of vitamin D supplements may be used to ameliorate deficiency in children with BA, some patients still fail to achieve normal 25(OH)D levels with oral supplementation. This is likely due to the markedly reduced expression of CYP2R1 in children with BA (Sun et al., 2021). For patients who respond poorly or not at all to oral vitamin D, sunlight exposure may offer a reliable means of stimulating endogenous vitamin D synthesis. However, in those with failed KPE who require hospitalization, oral supplementation with 25(OH)D appears to be a reasonable option for correcting vitamin D deficiency and preventing rickets (Daum et al., 1976).

Taken together, these findings suggest that vitamin D supplementation represents a novel therapeutic strategy for children with BA. However, it has yet to be systematically evaluated, and further clinical trials are warranted to confirm its efficacy (Bezerra et al., 2018; Hou et al., 2025).

In summary, bile acids, the gut microbiota, and vitamin D all have potential diagnostic value and may serve as potential therapeutic targets for children with BA. However, evidence for any specific component that can act as a surrogate marker reflecting perturbations across the entire axis in children with BA is currently lacking. From a therapeutic perspective, combined use of these three components might offer better efficacy than any single intervention in children with BA, although supporting evidence is also lacking.

## 8 Future perspectives

Over the past decade, research in children with BA has largely focused on alterations in the gut microbiota, bile acid metabolism, or vitamin D levels in separate contexts. However, the potential role of the bile acid-gut microbiota-vitamin D axis in the pathogenesis of BA has yet to be systematically defined. This emerging positive feedback loop offers novel insight for future investigation, but many key questions remain to be addressed.

As a multifactorial disease, research on BA must consider confounding factors beyond the variables of interest and adopt a more holistic approach to elucidate the role of the bile acid-gut microbiota-vitamin D axis in the disease. Given that the incidence of BA varies with geographic location and ethnicity, differences in study populations may contribute to heterogeneity in findings. Current data on bile acid profiles, gut microbiota alterations, and vitamin D levels in BA are derived mainly from populations in China, the United Kingdom, Indonesia, the Netherlands, and Austria. The lack of systematic data from other regions and ethnic groups may bias conclusions and limit the generalizability of existing findings (Hou et al., 2025). Future efforts should therefore include multicenter studies across diverse countries and ethnicities to further evaluate the applicability of current findings across populations.

Heterogeneity in experimental designs may also influence research results. Confounding factors such as dietary patterns, lifestyle and medication use may affect the gut microbiota composition and vitamin D levels in children with BA, thereby compromising the objective interpretation of the results. Consequently, further research is required to investigate the potential mechanisms between the gut microbiota composition and vitamin deficiency in children with BA (Hou et al., 2025).

Furthermore, due to differences in species between animals and humans, research data derived from animal models cannot be fully extrapolated to humans (Hou et al., 2025). Many clinical studies have limited sample sizes, and their findings are not entirely reliable (Li et al., 2017; Wu et al., 2024). Future studies should employ longitudinal, interventional, or prospective designs with large sample sizes and multicenter collaboration to further investigate the causal relationships and specific molecular mechanisms involving the bile acid-gut microbiota-vitamin D axis in BA (Thomas et al., 2020).

Meanwhile, current approaches for gut microbiota analysis rely primarily on 16S rRNA gene sequencing and metagenomic sequencing. However, these two methods may not accurately reflect the state of the microbiota in the gut (Boughanem et al., 2023). Moreover, most studies use faecal samples, with only a few utilizing intestinal contents, which may limit the completeness and depth of gut microbiota analysis (Boughanem et al., 2023).

Additionally, most studies on bile acid profiles in children with BA have focused on serum and faecal bile acid profiling. However, as the liver is the sole organ responsible for bile acid synthesis, it remains largely unexplored due to the difficulty of obtaining tissue samples (Zhang et al., 2022). Future research should therefore include a comprehensive analysis of bile acid metabolism in liver tissue, in order to fully elucidate the synthesis, transport, metabolism and functional regulation of bile acids in children with BA. This would offer a deeper insight into the complex interplay between bile acids, gut microbiota and vitamin D in this disease (Miao et al., 2023). It is worth noting that there has been little systematic research into changes in bile acids and gut microbiota in children with BA after LT. The characteristics of the dynamic changes during this phase and the mechanisms underlying their interactions remain to be elucidated.

Ultimately, the aim of the research is to translate the bile acid-gut microbiota-vitamin D axis into clinical practice for children with BA. However, non-invasive biomarkers and diagnostic tools for BA are still facing challenges related to insufficient validation and a lack of standardisation (Perez-Diaz-Del-Campo et al., 2023). Therefore, future clinical trials should consider combining multiple biomarkers to improve the accuracy of BA diagnosis (Perez-Diaz-Del-Campo et al., 2023). In terms of treatment strategies, while some patients respond poorly or not at all to UDCA or OCA, studies combining probiotics with standard UDCA and OCA therapies have demonstrated their efficacy (Harms et al., 2018; Yu et al., 2023). Furthermore, studies have indicated that combined supplementation with probiotics and vitamin D is more effective than either agent alone (Abboud et al., 2020). Clinical trials have also shown that concurrent supplementation with probiotics, such as *Bifidobacterium* and *Lactobacillus*, alongside vitamin D, results in greater increases in circulating vitamin D concentrations in patients (Raygan et al., 2018). It is therefore crucial to develop personalised, combined supplementation strategies for children with BA that take into account changes in bile acids, gut microbiota and vitamin D. Furthermore, long-term clinical studies are required to investigate the optimal dosage, duration and efficacy of these treatment strategies, as well as their durability of effects and clinical significance. It is worth noting that no dietary supplement should replace fundamental dietary adjustments and lifestyle interventions (Bellerba et al., 2021).

## 9 Conclusion

BA is a fatal neonatal cholangiopathy. Its aetiology is complex, and diagnosis and treatment remain significant challenges. Extensive research has suggested that bile acid metabolism, gut microbiota and vitamin D play crucial roles in the pathogenesis of biliary and hepatic diseases. This review systematically summarises the progress of research on these three factors in BA, proposing a potential theoretical framework centred on the bile acid-gut microbiota-vitamin D axis. The axis offers a deeper insight into disease pathogenesis by integrating their interplay. We summarise the dynamic characteristics of bile acid profiles, gut microbiota composition and vitamin D levels at different stages of the disease in children with BA. These three components create a positive feedback loop via the gut-liver axis, creating a vicious cycle that may drive disease progression and collectively exacerbate cholestasis, liver damage and liver fibrosis in children with BA.

This axis offers the potential for the early diagnosis of BA in children through a combination of non-invasive biomarkers, including characteristic bile acid profiles, key microbial abundance ratios, and vitamin D levels. This could lead to transformative treatment options for these children, involving strategies such as bile acid receptor agonists, probiotics and vitamin D supplementation.

However, in-depth research into the complex role of this axis in the pathogenesis of BA remains limited. The existing evidence is largely observational and based on small-sample studies, providing limited causal evidence. Furthermore, issues such as population heterogeneity, methodological limitations, and a lack of long-term clinical data persist. This makes it challenging to draw any definitive conclusions regarding the decisive role played by this axis in the disease.

These limitations should encourage scientists to utilise modern techniques, such as omics approaches, to explore the positive feedback loop mechanism between these three components in greater depth. In the near future, diagnostic and therapeutic strategies based on this axis show great promise. Future efforts should focus on large-scale, multicentre studies and randomised controlled trials to elucidate the underlying mechanisms of this positive feedback loop. The aim is to provide new insights into early diagnosis and a theoretical foundation for developing targeted intervention in BA. Concurrently, it is necessary to systematically evaluate the optimal dosage, duration of treatment, duration of efficacy and clinical safety of combined intervention, such as those targeting bile acid metabolism, modulating the gut microbiota and supplementing vitamin D. It is hoped that this will provide novel therapeutic options for reducing the incidence and improving the prognosis of children with BA.
